# Exploring the perceptions of Chinese adults toward overweight and obesity: A systematic literature review

**DOI:** 10.1111/obr.13913

**Published:** 2025-02-18

**Authors:** Yixi Wang‐Chen, Hui Yang, Nicole J. Kellow, Tammie S. T. Choi

**Affiliations:** ^1^ Department of Nutrition, Dietetics & Food Monash University Melbourne VIC Australia; ^2^ Department of General Practice Monash University Melbourne VIC Australia

**Keywords:** Chinese, obesity, perception, weight management

## Abstract

**Introduction:**

The prevalence of obesity among the Chinese population has increased more than three‐fold over the last twenty years. It is crucial to understand Chinese people's perceptions toward obesity to inform effective weight management initiatives. This bilingual systematic review aimed to synthesize the existing literature regarding the perceptions of Chinese adults toward overweight and obesity and provide insight on methodological implications and future research directions.

**Method:**

Six databases were searched from inception to 8th January 2025. Studies were included if they were published in English or Chinese, investigated perceptions toward overweight and obesity, and focused on Chinese adults living in or outside of Mainland China. Thematic synthesis was employed for data analysis.

**Results:**

Fifty‐three studies (24 in English, 29 in Chinese) were included, involving 83,688 participants. Three themes were identified; (1) Chinese adults connected obesity with appearance more than with health, (2) Chinese adults lacked practical knowledge to manage obesity, and (3) living with obesity was perceived as a solitary journey.

**Conclusion:**

Most studies were conducted on Chinese people within the healthy weight range, who predominantly focused on appearance‐oriented weight perception. There was a strong motivation for weight control, but a lack of practical weight loss strategies among Chinese adults.

**Practitioner applications:**

- The lack of practical weight management knowledge and weight loss failures lead to low self‐efficacy, which may be mistaken as low motivation for weight management among Chinese adults. However, Chinese adults generally have the motivation to control their weight. It is important to empower Chinese people living with overweight or obesity with practical skills and increase self‐efficacy through a multidisciplinary and affordable approach.
- The psychological burden caused by obesity stigma and the influence of Chinese culture makes the weight management journey lonely and challenging for Chinese adults living with overweight and obesity. Practitioners may need to openly address these issues and help reduce the mental burden toward more effective weight loss interventions.

**Abbreviations**
BMI: Body Mass Index
PRISMA: Preferred Reporting Items for Systematic Review and Meta‐Analysis
CASP: Critical Appraisal Skills Programme
AXIS: Appraisal tool for Cross‐sectional Studies
RoB 2: Cochrane Risk‐of‐bias Tool for Randomized trials Version 2
WC: Waist Circumference
HWR: Healthy Weight Range

## 1 INTRODUCTION

The escalating prevalence of overweight and obesity has become a global public health concern across both developed and developing countries. The Chinese population (encompassing individuals of Chinese nationality, 中国人, as well as those of Chinese ethnicity, 华裔, in this paper, collectively referred to as 华人) as one of the major demographics in the world, is not exempt from this trend. Predominantly residing in Mainland China, this population has witnessed a significant rise in obesity rates over the last 20 years.^1^ Cross‐sectional surveys conducted in Mainland China show that the obesity rate increased from 4.2% in 1993 to 15.7% in 2015, with parallel incremental increases observed in abdominal obesity from 20.2% to 46.9%.^1^ A similar upward trajectory has also been found in Taiwan (from 16% to 20% and 27% to 47% for obesity and abdominal obesity rates respectively between 1996 and 2016).^2^ Furthermore, a large cross‐sectional study conducted in Mainland China involving 15.8 million adults revealed that in 2023 one in every two Chinese adults was categorized as living with overweight or obesity.^3^ Multiple studies have indicated that Chinese individuals face a higher health risk at comparable Body Mass Index (BMI) levels to Caucasians. For instance, a large cohort study suggested that Asian individuals, including Chinese people, classified as overweight exhibited a significantly higher risk of all‐cause mortality than their Caucasian counterparts, when using the same BMI classification.^4^ Additionally, a cross‐sectional study in Australia assessed cardiovascular disease risk factors among older participants, revealing that people born in Southeast Asia were more susceptible to developing diabetes, hypertension, and hypercholesterolemia at a relatively lower average BMI.^5^ The escalating prevalence and health risks of obesity among Chinese people underscores the urgency for developing effective interventions.

People’ s perception toward obesity is likely to play an important role in influencing their weight management behavior.^6^ It is not uncommon to see that the subjective perception of an event or situation exerts a greater influence on an individual's actions than the objective reality does. For instance, perceived social status has been found to have a stronger association with health‐compromising behaviors than actual social status.^7^ Similarly, how individuals perceive their weight status serves as a more reliable predictor of their weight loss efforts than their actual weight does.^8^ Gaining insight into individuals' perception toward obesity is a key step for developing effective weight management intervention programs.^9^ For Chinese people, the unique Confucianism ideology, collectivist society, and modern mass media information exposure may have a profound influence on how Chinese people develop their perceptions about weight issues.^10^, ^11^ It is therefore valuable to understand Chinese people's obesity‐related perceptions as this may inform more culturally appropriate interventions for weight management.

” Perception” is a concept with broad meaning. According to the Oxford Dictionary, it is defined as “an idea, a belief or an image you have as a result of how you see or understand something”.^12^ Similarly, the Cambridge Dictionary describes it as “a belief or opinion, often held by many people and based on how things seem”.^13^ From a psychological standpoint, perception is a process through which individuals make sense of the world by actively selecting, organizing, interpreting, and evaluating incoming information.^14^ These definitions suggest that perception can be understood both as a process and as the outcome of subjective consciousness. Interestingly, the term “perception” has rarely been used in behavioral theories but the relevant concept of “perception” has been utilized extensively. For instance, the Health Belief Model refers to the term “perceived”; The Transtheoretical Model speaks of “contemplation”; the Social Cognitive Theory mentions “expectations” and “self‐efficacy”; and the Self‐determination Theory discusses “motivations”. Despite their nuanced differences, these terms share a common psychological and linguistic root with “perception”, making it an encompassing term that can unify these diverse concepts without contradiction.^15^

This review aimed to systematically explore research findings in English and Chinese publications to understand the perceptions of overweight/obesity among Chinese (华人) adults living in and outside of Mainland China. Its primary objective was to synthesize the prevailing perception themes from existing evidence and identify research gaps. It also sought to evaluate the research methodology employed in existing studies, while suggesting directions for future research.

## 2 METHOD

### 2.1 Data sources and search strategy

This review was conducted to comply with the 2020 Preferred Reporting Items for Systematic Review and Meta‐Analysis (PRISMA) expanded checklist.^16^ The review proposal was registered on the International Prospective Register of Systematic Reviews on 23 Jun 2023 (CRD42023435348). Six databases (4 English databases, and 2 Chinese databases) were selected to encompass a variety of disciplines including medicine, psychology, and social science. The English databases were *Medline (*via *Ovid), APA PsycINFO (*via *Ovid), Social Science Premium Collection (*via *ProQuest*), and *Scopus*. The Chinese databases were the *Chinese Journal Database in Wanfang (万方, covering simplified Chinese written papers published in Mainland China, Hong Kong& Macao Special Administrative Regions)* and *Chinese Electronic Periodical Services in Huayi Online Library (covering traditional Chinese written papers published in Taiwan)*. Papers written in either traditional or simplified Chinese were included. The initial search was conducted to retrieve relevant articles published from the database inception to 17 July 2023. A*n updated search was conducted across the six databases to include papers published from 17th July 2023 to 8th January 2025 using the same search strategy. A total of 1122 articles were identified, of which 5 additional papers met the inclusion criteria after dual screening. All data was updated accordingly*.

In order to avoid the limitations that may arise from the use of more specific terminology, “perception” in this paper is conceptualized as the “mental representation of the world,”^17^ serving as a comprehensive term for numerous mental concepts, including belief, motivation, attitude, desire, knowledge, and intention. The English searching keywords were (“Chinese” OR “China” OR “Taiwanese”) AND (“obesity” OR “overweight” OR “body weight” OR “body mass index” OR “body image” OR “body size” OR “fatness”) AND (“perception” OR “perspective” OR “attitude” OR “opinion” OR “belief” OR “sentiment” OR “weight/fat prejudice/stigma/bias/discrimination” OR “knowledge” OR “comprehension” OR “awareness” OR “self‐perception/esteem/confidence”). Accordingly, Chinese searching keywords were translated and discussed among three Chinese‐speaking researchers within the team. The Chinese keywords were (“肥胖“OR “超重” OR “体重” OR“体重指数” OR “体质指数” OR “身体质量指数” OR “体型” OR “体形” OR “体脂” OR “身体形态” OR “身体意象”)AND (“观点” OR “态度” OR “看法” OR “信念” OR “歧视” OR “污名” OR “偏见” OR “知识” OR “理解” OR “自我感知” OR “自我认知” OR “自尊” OR “自信”). The full database search strategy is provided in Supplementary Information Table S1.

### 2.2 Study selection

All retrieved citations were imported into Covidence (Covidence Systematic Review Software, Veritas Health Innovation, Melbourne, Australia) for screening, with title and abstract screening followed by full‐text screening conducted independently by two reviewers. Disputes were resolved through discussion within the team. Papers that met the following eligibility criteria were included: (1) the research sample included Chinese adults (≥18 years) living in China and other countries, with any health condition. (2) the phenomenon of interest was perception toward overweight and/or obesity; (3) any research data collection methodology e.g., survey, interview, intervention, or longitudinal research methods; (4) participants' perception, beliefs, attitudes toward obesity and its management were collected; (5) all types of original research study designs, including qualitative, quantitative or mixed method studies. (See the detailed eligibility criteria in Supplementary Information Table S2).

### 2.3 Data extraction and quality assessment

A data extraction table was developed to extract research aims, publishing year, funding source, data collection method, sample characteristics, and main qualitative outcomes related to obesity perceptions. When a study explored the perceptions of both Chinese health professionals and the general Chinese adult population, only the general Chinese adults' perceptions toward overweight and obesity were extracted. Three quality appraisal tools were chosen for their appropriateness. All of the qualitative studies were assessed using the Critical Appraisal Skills Programme (CASP) Qualitative Studies Checklist.^18^ This tool assesses three domains of a study via 10 questions, to determine (a) if the results are valid (b) what the results are, and (c) if the results can be applied locally.^18^ The included quantitative cross‐sectional studies were appraised by the Appraisal tool for Cross‐sectional Studies (AXIS).^19^ This tool identifies potential biases within studies based on a set of 20 questions, including study design, target population, sample size justification, sampling frame, sample selection, non‐responders, measurement validity, and reliability, statistics, internally consistent results, etc.^19^ The quantitative interventional studies were assessed via the Cochrane risk‐of‐bias tool for randomized trials version 2 (RoB 2).^20^ The RoB 2 tool evaluates a set of five domains within a study, resulting in a study being determined to have either a “low risk,”, “some concerns,” or a “high risk” of bias. The five domains assess bias arising from the randomization process, deviations from intended interventions, incomplete outcome data, outcome measurement bias, and selective reporting of results.^20^ The first author (YW) extracted data from all included papers and appraised their quality or risk of bias. The remaining authors conducted data extraction and quality assessment on a subset (33%) of papers that were randomly selected. Data extracted from all the Chinese written papers were translated into English. The results were compared and disagreements were resolved by discussion.

The research team contained three bilingual researchers who were English and Chinese‐speaking (YC, HY, TC) and one monolingual English‐speaking researcher (NK). Citation screening, data extraction and quality assessment of English language papers was shared among all authors, whereas screening, data extraction, and quality assessment of the Chinese language papers was shared among the three bilingual researchers. The research team communicated in English and all data analysis and synthesis was conducted in English.

### 2.4 Data analyses

Thematic synthesis was employed on the extracted results from the included studies, as this method preserves an explicit and transparent link between the results of the primary studies and the synthesized outcomes of the review.^21^ Data were analyzed using an inductive thematic analysis method^22^ whereby findings from each study were open‐coded, before grouping codes into similar concepts or themes to address the research questions. Analysis was assisted by QSR‐Nvivo (Version 14.23.0). To enhance research rigor, investigator triangulation was adopted. A subset of data (20%) was analyzed independently by another author (TC or HY). Authors then came together to discuss their independent findings and build on individual interpretations of the data.

## 3 RESULTS

**FIGURE 1:**
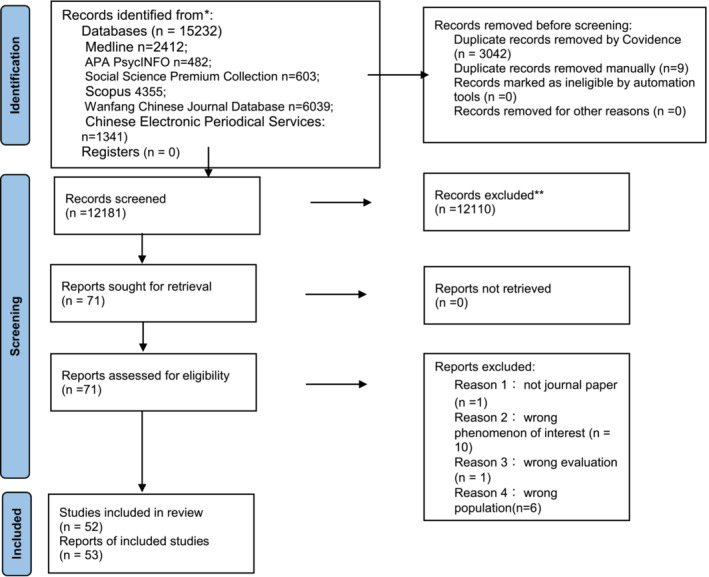
PRISMA flowchart showing the study identification and screening process.

A total of 15,232 records were screened with 53 eligible studies included for review (see Figure 1 for the PRISMA flowchart). All studies were published between 1999 and 2024. There were 24 English papers and 29 Chinese papers (22 were written in simplified Chinese, 7 were in traditional Chinese). Nine articles were qualitative papers, and 44 were quantitative papers (43 cross‐sectional studies and 1 interventional study). Given two papers reported the same study based on different perspectives, there were 52 studies in total. The articles originated from different regions; 32 from Mainland China, 14 from Taiwan, 3 from the USA, 1 from Singapore, and 2 as cross‐nation studies. The majority of participants were from urban areas and only two studies^23^, ^24^ included participants from rural areas. For studies involving participants living in other countries, only one study recruited some Chinese immigrants as part of the sample,^25^ with the remaining studies engaging local residents with Chinese ethnicity or nationality (see Table 1 for Study details and qualitative outcomes).

**TABLE 1:** Study details and qualitative outcomes.

First author and year	Study type	Research method	Participants' characteristic	Study used BMI threshold (kg/m^2^)	Main findings
Chang et al 2004^26^	Qual	Pilot study; Purposive sampling; In‐depth interview; content analysis.	All F, n = 5, university students majoring in nursing, medicine, and life science; age range: 20–23 y, mean age 21.6 y; all were within healthy weight range; all considered themselves as overweight and had previously used weight control methods.	healthy weight: 19.8–24.2	1. Social labeling of the overweight: a slim image was overwhelmingly preferred; 2. Feeling sad and inferior because of being overweight. 3. Pursuing a body weight that meets a standard that is far below what is considered a healthy standard; 4. Self‐restraint for weight reduction: a constant source of stress and an endless struggle. 5. Mood went up and down with weight loss and gain; 6. Other people's (family members, friends, classmates, clothing sales, strangers, acquaintances) comments were important to the participants; 7. There was a plethora of ubiquitous stimuli for them to lose weight (other people's teasing, jokes, mass media, classmates' weight status; 8. They felt dejected due to their body size when purchasing clothes; 9. Physical appearance and attraction were the most important motivation for their weight control; 10. Pursuing a slender body was intended to earn social approval; 11. Feeling sad and inferior due to being overweight; 12. They knew that their weight was within normal range, but the weight standards for health and beauty were different.
Chang et al. 2021^27^	Qual	Purposive sampling, In‐depth interview, thematic analysis	N = 32 (M: n = 14, F: n = 18), participants involved a WeChat‐based weight control program; age range 21–53 y, mean age 35.6 ± 7.7 y; all previous BMIs were above 25, post BMI 17.9–47.9 kg/m^2^.	no information	1. The doctors' help via WeChat made participants feel: knowledge infusion, efficacy enhancement, timely feedback, emotional support; 2. WeChat surveillance had positive influence (self‐awareness) on some people but negative effect (invasive technology)on others; 3. Peer support fostered empathy and a sense of belonging, and had a mutually reinforcing relationship with peer comparison and peer‐based surveillance; 4. Peer comparison enhanced motivation and positive competition. However, it also reinforced negative group norms, and resulted in downturns in reference standards and collective inactivity; 5. Empathy, companionship, mutual encouragement, and collective empowerment were intangible resources.
Cheng et al 2018^28^	Quant Cross‐sectional study	Convenience sampling; four validated tests (beliefs about person with obesity scale, everyday discrimination scale, three‐factor eating questionnaire‐revised 18‐item version, the hospital anxiety and depression scale); self‐reported BMI.	N = 400 (M: n = 175, F: n = 225), university students in Hong Kong; mean age 20.2 ± 1.5 y; mean BMI 20.6 ± 3.2 kg/m^2^； 15.3% were overweight or obese； 21% self‐perceived their weight as overweight or obese.	≥23 overweight <23 non‐overweight	1. Weight bias or perceived weight stigma was associated with psychological distress regardless of actual or perceived weight status; 2. Self‐perceived and actual overweight groups were likely to have weight bias, accept negative attitudes toward people with obesity, and develop depression and anxiety; 3. Perceived weight stigma was more strongly correlated with emotional eating in the self‐perceived overweight group; 4. In the group that was not overweight, eating disturbance was associated with stronger belief that obesity can be controlled. They also valued a slender figure. They had a higher weight bias and lower perceived weight stigma score (they felt they experienced less stigma).
Cheng & Zhang 2017^29^	Quant Intervention study	Researchers gave the intervention group a task to imagine themselves as living with obesity first, then take the Implicit Association test. The control group took the same test without any prior intervention.	N = 80 (F: n = 40, M: n = 40), university students. No other information provided.	no information	1. All participants had a negative implicit attitude towards people with obesity; 2. The intervention did not significantly change the negative attitude but reduced it somewhat.
Chu, Yu‐Wei 2008^30^	Qual	In‐depth interview, using a semi‐structured interview guide; no information about sampling method; thematic analysis; investigator triangulation.	All F, n = 6, university students; healthy weight; BMI < 24 kg/m^2^. no other demographic information provided.	healthy weight.: BMI < 24	1. Participants wanted to find an effortless weight loss method, no exercise, no dieting; 2. Monitoring weight was a tedious and anxiety‐provoking daily activity; 3. They considered weight control as hard and miserable work; 4. They did not want other people to know that they were undertaking weight management; 5. They used calculations or comparison with others to define their ideal weight; 6. Weight loss was the priority; body shape was a secondary priority; 7. They felt ashamed or embarrassed when wearing bigger size clothes; 8. They thought being fat is ugly, cannot wear beautiful clothes, giving other people bad impression; 9. Being criticized by closer friends, boyfriends, or family members is hurtful. 10. They believed that being thin will leave a good impression for others and increase self‐confidence; 11. They believed the society pushes females to be thin. The standard was stricter for females than males.
Fan& Che 2023^31^	Quant Cross‐sectional study	Random cluster sampling in Jiangsu Province; no information on sample size calculation; used Anti‐obesity Attitudes Test and Weight Bias Internalization Scale; Response rate of valid questionnaires: 95.7%; self‐reported BMI; Unclear data analysis method.	N = 201 (M: n = 63, F: n = 138), mean age 23 y; underweight n = 45, healthy weight n = 130, overweight n = 26	18.5–23.9 healthy weight, 24–27.9 overweight, ≥28 obesity	1.More male than female students held strong negative attitudes toward obesity.; 2.Those with low or normal weight had stronger negative attitudes toward obesity than those who were overweight.
Fu et al 2011^23^	Quant Cross‐sectional study	Secondary study, used data from a survey conducted during 1999–2002 in Taiwan; self‐developed questionnaire done by interview; Hannemann's welfare evaluation model.	N = 578 (M: n = 208, F: n = 370), villagers in Taiwan; mean age = 45.8 ± 14.4 y；mean BMI 25.6 ± 3.7 kg/m^2^； 30% of sample were living with obesity.	<24 healthy weight, 24–26 overweight, ≥27 obese	1. 79% indicated that obesity affected their work achievement; 2. 60% had tried to control weight; 3. Female respondents with obesity had a higher WTP for weight loss therapy, males without obesity had a relatively low WTP; 4. Average WTP amount was 362US$; 5. Those who felt obesity may affect work achievement and who felt obesity may affect social relations were found to have a greater WTP; 6. Higher education, higher income females were more likely to pay higher amount for weight reduction therpies; 7. Female respondents would be willing to pay US$214 more than a male respondent. A respondent with obesity was willing to pay US$128 more than a respondent without obesity.
Hao et al. 2023^32^	Quant Cross‐sectional study	Convenience sampling, recruited participants in one of the universities in Ganzhou City, Jiangxi province in China. Validated questionnaires (the Chinese version of the Dutch Eating Behavior Questionnaire, The Physical Activity Rating Scale‐3), Instrument‐measured BMI; Independent sample t‐test, Pearson's chi‐squared test, Tukey's test, Multiple regression analyses.	N = 1714 (M: n = 933, F: n = 781); All were university students; age range 18–24 y, mean age 19.8 ± 2.0 y;	18.5–24.9 healthy weight, >25 overweight, ≥30 obesity	1. The average level of body dissatisfaction was significantly higher in female than male university students.
Huang et al 2001^33^	Quant Cross‐sectional study	Stratified random sampling; self‐developed questionnaire.	N = 645 (M: n = 246, F: n = 399), adult residents living in Guangzhou city; all were with overweight or obesity; education level, middle school education or below 30%; high school education 35%, college and above 35%.	female: overweight or obesity BMI > 23; male overweight or obesity BMI > 25	1. 34.1% believed obesity does not harm health, and has no relationship with chronic diseases; 2. 62.9% believed people will put on weight as they age; 3. 7.9% thought the most long‐term effective weight control method was weight loss medicine; 4. Preferred information sources: 55% chosen TV & newspaper, 26% chosen professionals; 5. Weight control methods: 70% chosendiet and exercise, 8% chosen weight loss medicine, 14.9% chosen liposuction, qigong, acupuncture; 6. 72.1% were not happy with the result of their weight loss efforts.
Hu et al. 2007^34^	Quant Cross‐sectional study	Convenience sampling from a hospital fitness program; instrument‐measured BMI; self‐developed questionnaire.	all F, n = 96, 50 were living with obesity, 46 were within healthy weight range; age range 20–59 y, 65% were aged 20–39 y.	18.5–24 healthy weight, 24–27 overweight, ≥28 obesity	1. About 62%–84% of both groups strongly agreed with three negative statements (people with overweight are clumsy, lazy, and unhealthy); 2. More in the group with obesity agreed “employer should not hire people with overweight” than the group with healthy weight; 3. 94% of the group with obesity strongly agreed with “people with excess weight are physically unattractive”, while 78% of the group with healthy weight agreed with this point; 4. Both groups tended to reject obesity for aesthetic rather than medical reasons; 5. The group with obesity showed less control over portion size, fried foods, and sugar‐containing beverages; 6. The group with obesity had higher score on “you should cerebrate or award you by eating” than the group with healthy weight; 7. 47% had inaccurate image of their weight status.
Ji et al. 2024^35^	Quant Cross‐sectional study	Participants were recruited via in person (70%) and online method (30%); Self‐developed survey; Justified sample size. Self‐reported BMI, Descriptive statistics method. (Only the general Chinese adults' perceptions were extracted for this review)	N = 7000 (M: n = 3543, F: n = 3457), all BMI ≥ 28, mean BMI 30.6; Mean age 43.2 (18.0–84.0) y; 33.6% of the sample had no self‐reported comorbidity, 22.3% had one, 19.3% had two, 14.3% had three, 10.7% had four and above comorbidities.	18.5–23.9 healthy weight, 24–27.9 overweight, ≥28 obesity	1. 76.8% of participants saw obesity as a chronic disease, but 30% did not recognize they had it. Over 80% had no plans to lose weight in the next month; 2. 38% rated their general health as good; 3. 74.3% had health concerns if they maintain current weight. 4. 74.7% believed weight loss was entirely their own responsibility; 5. Top weight loss motivations: improving fitness, feeling better, and boosting confidence; 6. Median weight loss target was 16.7%, while the recommended goal was 10%; 7. Few viewed medical treatments as effective; 8. Common reasons for weight regain: struggling to follow diet/exercise plans and staying motivated; 9. Family/friend support and strong motivation were seen as key success factors; 10. Only 19.1% found discussing weight management with a health professional helpful.
Jiang et al 2017^36^	Quant Cross‐sectional study	Convenience sampling in a private university; justified sample size; used three validated tests (Implicit Association Test, attitudes toward person with obesity, behavioral intentions toward overweight and obesity).	all F, n = 104, university students in Singapore; cultural identity: 70.2% Chinese, 16.3% Indian, 1.9% Malay, 11.6% others; age range: 18–44 y, mean age 21.6 ± 3.3 y; BMI range: 14.9–33.3; mean BMI 21.4 ± 3.9 kg/m^2^; underweight 22.1%, healthy weight 64.4%, overweight 8.7%, obese 4.8%.	18.5–24.9 healthy weight, 25–30 overweight, ≥30 obesity	1. Asian female participants exhibited a strong implicit anti‐fat bias, but did not report this bias explicitly; 2. Implicit anti‐fat bias toward individuals with overweight and obesity was widespread and strong in Asian female participants.
Jin et al 2015^37^	Quant Cross‐sectional study	No information about sampling method; questionnaire was developed by China Health Education centre. Instrument measured BMI.	N = 323 (M: n = 161, F: n = 162), governmental workers; age range: 18–45 y, (21–30 y: 25.1%, 31–40 y: 49.9%, 41–45 y: 25.1%); underweight 12.1%, healthy weight 63.8%, overweight 19.2%, obese 5.0%.	18.5–23.9 healthy weight, 24–27.9 overweight, ≥28 obesity	1. 32.9% males and 33.3% females considered themselves as overweight. About half participants were not happy with their weight; 2. 16.2% males and 4.9% females did not consider obesity as a risk for health; 3. Reasons for obesity: lack of exercise 88.2%, over consumption of sweet food 81.4%, too much snacks 60.4%, gene 55.7%, too much staple food 31.3%; 4. Information sources: TV and radios 71.5%, book/magazine/book 60.1%, internet 50.5%, friend 28.5%; 5. Their weight monitoring methods: using weight scales 63.8%, clothes size 33.4%, health check 32.2%, from other people 14.9%; 6. Diet control and exercise were popular methods to lose weight.
Jin et al 2016^38^	Quant Cross‐sectional study	Convenience sampling method, chose workers from two companies in Chengdu city; questionnaire was developed by the China Health Education Centre.	N = 337 (M: n = 172, F: n = 165), enterprise management workers, age range 18–45 y; 21–31 y (32.1%); 32–38 y (34.4%), 39‐45y (33.5%); underweight 7.7%, healthy weight 60.5%, overweight 26.1%, obese 5.3%。 Central obesity 41.3% for male, 24.9% for female. 14.7% had healthy BMI but central obesity.	18.5–23.9 healthy weight, 24–27.9 overweight, ≥28 obesity	1. Reasons for obesity: lack of exercise 83.4%, overeating sweet food 77.8%, snacking 68.8%, irregular meals 46.0%, genes 40.7%; 2. Information sources: TV/radio 66.5%, book/magazine/newspaper 49.3%; internet 47.2%, friend 27.9%; 3. 80.7% were confident with their weight control methods; 4. 42.4% were unhappy with their weight; 5. 10.4% of people with overweight had not thought about weight control; 6. Motivation for weight control: 45.4% health consideration, 37.4% appearance, 34.4% negative impact on life; 7. Majority did not think obesity or overweight represented a good life and attractiveness; 8. Preferred method of weight reduction: 68.8% chosen exercise, 61.4% chosen diet control; 9. 14.0% knew how to calculate BMI correctly.
Kazuma Sato 2021^39^	Quant Cross‐sectional study	Secondary study; used data from a survey conducted in 2009–2013; multi‐stage sampling method; Survey was completed by face‐to‐face interview; subjective welling‐being was measured by the question “overall, how happy would you say you are currently?”; econometric model.	N = 1380 (M: n = 690, F: n = 690), residents living in 6 major cities; age range 20‐69y, mean BMI 23.2 for the Chinese sample, mean BMI 28.4 for the US sample.	18.5–24.9 healthy weight, >25 overweight, ≥30 obesity	1. Chinese men with overweight or obesity and women with overweight were happier than those who were within the healthy weight range. In US, there was no association between BMI and happiness; 2. Positive impact of obesity was more pronounced in men than women in China. A positive association between being underweight and happiness was mainly found in American women.
Keyser‐Verreault 2022^40^	Qual	Data was drawn from author's previous ethnography study conducted between 2014–17 in Taipei. Purposive and snowball sampling, In‐depth interviews, thematic analysis	N = 62, All F, All hold master's and above degrees. All identified themselves as middle class; unmarried n = 13, married with no children n = 14, married with children n = 35; Age range 29–39 y; Almost all lived in capital city.	no information	1. Many felt deeply hurt when fat‐shaming came from their mother; 2. Holidays and family events were particularly difficult for participants being overweight; 3. Female beauty, especially thinness is seen as key to women's embodied capital; 4. Mothers often enforce strict body discipline, sometimes via humiliation on their daughters, in order to secure their daughter's future and maintain family face.
Lee et al. 2023^41^	Qual	Purposive sampling, focus group, thematic analysis	N = 13 (all F), pregnant women; age range: 20–36 y; their pre‐pregnancy BMI was above 25 at antenatal booking (range 25–36).	≥ 25 as overweight	1. Participants said there was no specific Mandarin Chinese app for gestational weight management; 2. Safely delivering a healthy baby was the main priority; 3. They wanted to know how much weight gain was appropriate and how to manage weight gain; 4. Feeling like a failure and having low self‐esteem because they could not control weight; 5. They had low self‐efficacy; 6. They thought incentives, reward systems (monetary and material rewards) on the app would be useful; 7. They preferred concise and easy‐to‐digest information; 8. Chinese culture encourages them to eat more; 9. They felt lonely thus wanted community network and family support; 10. They wanted a user‐friendly app, as a credible information source and venue for peer encouragement; 11. Struggling with life and desiring peace of mind.
Li et al 2019^42^	Quant Cross‐sectional study	Cluster stratified random sampling in three universities in Haikou city; self‐developed and validated questionnaire (Content validity index 0.82, Cronbach's alpha 0.73); instrument measured BMI	N = 632 (M: n = 290, F: n = 342), University students; mean age 20.4 ± 2.2 y; mean BMI 20.7 ± 3.0 kg/m^2^; healthy weight.: 72%, Overweight 6%, obese 3.8%.	18.5–23.9 healthy weight, 24–27.9 overweight, ≥28 obesity	1. 39.7% of students misjudged their weight status. They considered themselves as overweight despite being within a healthy weight range; 2. 53.3% used calculation method to assess their weight; 10.1% relied on other's view; 3. 81.2% knew the causes of obesity; 77.4% knew the risk of obesity; 4. Majority students did not lead a healthy lifestyle. Midnight snack, having soft drinks, staying up late, ordering takeaway were common.
Lin et al 2021^43^	Quant Cross‐sectional study	Proportional stratified sampling in a university; justified sample size; three published questionnaires (weight self‐stigma questionnaire, body areas satisfaction scale, media influence questionnaire); self‐reported BMI.	N = 963 (M: n = 276, F: n = 678), university students; age range: 20–35 y, mean age 21.8 ± 4.4 y; mean BMI for females 20.7 ± 3.1 kg/m^2^, Males 22.4 ± 4.2 kg/m^2^.	no information	1. Females had a higher intention than males to control their weight, a higher perception of weight self‐stigma, and were more dissatisfied with their weight than males; 2. Those with higher BMI, weight self‐stigma, body dissatisfaction and media influence had stronger weight control intention; 3. People with obesity were more likely to internalize weight stigma from public attitudes and influences, and were more dissatisfied than others with their body appearance; 4. Over 1/3 participants had weight loss intention.
Lin et al 2024^44^	Quant Cross‐sectional study	Secondary analysis. Data were drawn from the Adelphi Real World Obesity Disease‐specific Program conducted in China between April and July 2022. No detailed information was provided for the original data collection method. Self‐reported BMI, Descriptive statistics. (Only the general Chinese adults' perceptions were extracted for this review)	N = 801(M: n = 357, F: n = 444), Mean age 38.3 ± 11.7 y; All were enrolled in a weight management program. All BMI ≥ 28. Mean number of diagnosed comorbidities for the sample n = 1.9 ± 1.7;	≥28 obesity	1. 67.7% of participants with obesity‐related comorbidities started weight loss due to their comorbidities; 2. 63.4% aimed to lose at least 20% of their weight; 3. Participants wanted to lose weight for better quality of life and both physical and mental benefits; 4. The main reasons for stopping weight loss were difficulties in maintaining routines and slow progress; 5. 82% were dissatisfied with their current weight loss attempt.
Lin & Huang 2010^45^	Quant Cross‐sectional study	Stratified random sampling in 6 universities; self‐developed and piloted questionnaire; no BMI information; self‐reported weight status.	N = 1045 (F: n = 457, M: n = 588), university students.	no Information	1. Over 1/3 of female students considered themselves as overweight; 2. Students who considered themselves overweight preferred high‐fiber diets and calorie‐controlled diets.
Lin & Lin 2016^46^	Quant Cross‐sectional study	Convenience sampling, the questionnaire includes four sections (basic information, contour drawing raring scale, willingness of weight control, eating attitudes Test‐26). All are validated tests. Instrument‐measured BMI.	N = 509 (All F), university students, Mean BMI 20.9 ± 3.5 kg/m^2^.	no information	1. The participants' perceived current body weight was heavier than their desired body weight; 2. When their perceived body shape was heavier, desired body shape was thinner; 3. The more discrepancy between the desired and perceived body shape, the higher score of their weight reduction intention and eating attitudes; 4. Their willingness to undertake exercise had no relationship with weight control.
Liou & Bauer 2010^47^	Qual	Used Health belief model, the theory of planned behavior and social ecological models, purposive sampling, In‐depth interview, thematic analysis.	N = 40 (F: n = 24, M: n = 16), US‐born Chinese American descent, full time college students, 60%, employees 40%; age range 18–30 y, mean age 22 y.	no information	1. Encouragement to overeat: respondents felt traditional Chinese cultural pressures to overeat (do not waste food, better to be a little overweight), in conjunction with American emphasis on fast food and large portion sizes; 2. Collectivism versus Individualism: American culture promotes leadership and teamwork, whereas Chinese culture promotes conformity. There was pressure to maintain a healthy weight as obesity reflects poorly on the entire Chinese race; 3. Gender Factors: Women felt much greater pressure than men to be thin within both American and Chinese culture; 4. Social Norms: Respondents felt pressure to do what their peers do; 5. Chinese food practices were generally viewed as more healthful than American practices.
Liou et al 2007^48^	Qual	Used health belief model, the theory of planned behavior and social ecological models, purposive sampling, In‐depth interview, thematic analysis.	N = 40 (F: n = 24, M: n = 16), US‐born Chinese American descents, full‐time college students, 60%, employees 40%; mean age 22 y.	no information	1. 55% participants considered environmental factors to be major causes of obesity; 2. 40% believed genetic factors had a dominating effect in developing obesity; 3. 52% perceived the benefits of the protective Chinese genetic factor and healthful traditional Chinese diet; 4. 54% stated they were satisfied with their weight; 5. Believed healthful eating benefits: prevent obesity, healthy body, increase quality of life; 6. Believed barriers of healthy eating: social environment (celebrations, parties, eating with friends), physical environment (abundance of junk food), traditional Chinese culture encourage overeating, psychological factors (boredom, stress, reaction to deprivation); 7. 83% respondents indicated parents were major influence impacting their food choices; 8. Friends were also important social influence; 9. Healthy eating was believed to result in a favorable physical appearance, healthier skin, and a toned body; 10. Obesity was seen predominately as a non‐Asian phenomenon.
Liou et al 2009^49^	Quant Cross‐sectional study	Multi‐stage sampling, self‐developed questionnaire.	N = 1060 (F: n = 791, M: n = 214), adult patients in weight loss clinics; 18.5% were 18–24 y, 31.2% were 25–34 y, 25.3% were 35–44 y, 25% > 45 y; 16% healthy weight, 15.6% overweight with no co‐morbidity, 6.7% overweight with at least one co‐morbidity, 60.2% obese.	<24 healthy weight, 24–26 overweight, ≥27 obesity	1. Most respondents had unrealistic weight loss goals, 2/3 of subjects expressed a desire to lose more than 20% of their initial body weight； 2. Females tended to have more dramatic weight loss goals than males； 3. 45% of females wanted to lose as much as 30% of their initial weight; 4. Females expected to lose weight faster than males; 5. 22% of subjects wanted to lost weight at a rate of 6 kg/month； 6. Females were more likely than males to take anti‐obesity drugs, have inadequate physical activity； 7. Participants with higher BMI were more likely to take anti‐obesity drugs and have unrealistic weight loss goals than those with healthy weight; 8. Unrealistic weight loss goals were more common in younger subjects.
Liou et al 2014^25^	Quant Cross‐sectional study	Convenience sampling; self‐developed and validated questionnaire (Cronbach's alpha > 0.7); self‐reported BMI.	N = 300, (F: n = 195, M: n = 105), Chinese Americans; age range 18–40 y, mean age of 26 ± 6.8 y; culture identity: Western identified 117, bicultural 125, Asian identified 54; weight status: 67% healthy weight, 14% overweight, 5% obese, 9% underweight, average BMI 22.6 ± 3.8 kg/m^2^, ranging from 13.8 to 42.1. 63% college degree and above; 72% unmarried.	18.5–24.9 healthy weight, >25 overweight, ≥30 obesity	1. Bicultural participants had the highest percentage of overweight, Western identified participants had the highest percentage of obesity； 2. Participants believed adopting the following behaviors will bring benefit: exercising, limiting intake of high‐calorie soft drinks and juice, using less oils and fat in cooking； 3. Participants believed: a. unhealthy snack foods are cheaper than healthy snacks, b. it was convenient to eat pre‐packed, c. lack of time in preparing home‐cooking meals, d. eating healthily would need to spend more money; 4. Asian‐identified participants made more healthier eating behavior (more steamed food less junk food) than Western‐identified participants.
Liu et al 2015^50^	Quant Cross‐sectional study	Multi‐stage stratified random sampling; used questionnaire developed by China education centre; self‐reported BMI.	N = 1356 (M: n = 678, F: n = 678), civil servants 23.8%, university students 27.1%, company managers: 25%, institutional staff 24.0%; age range: 18–45 y, underweight: 11.8%, healthy weight.: 66.1%, overweight:19.9%, obese: 2.2%, central obese: 22.9%.	18.5–23.9 healthy weight, 24–27.9 overweight, ≥28 obesity	1. 70.4% did not know BMI; 2. 28.3% participants with overweight considered their weight as healthy; 3. Majority participants with obesity knew they carried excess weight; 4. 90% knew that obesity is harmful for health; 5. 86% thought that overweight and obesity influence social life; The main effect was that they could not wear beautiful clothes; 6. 73.4% considered obesity as a disease; 7. 93.7% believed that people with obesity should control their weight; 8. Weight loss methods: exercise, calorie control, it was rare that people wanted to seek help from professionals.
Ma et al 2011^51^	Quant Cross‐sectional study	Stratified cluster random sampling; self‐developed and validated questionnaire (Cronbach's alpha 0.892).	N = 1823 (all F), university students; mean age 19.9 ± 1.2 y； mean BMI 20.0 ± 1.9 kg/m^2^；underweight and healthy weight 97.3%.	18.5–23.9 healthy weight, 24–27.9 overweight, ≥28 obesity	1. 51.5% considered themselves to be living with overweight or obesity; 2. 67.6% thought about losing weight; 3. 45.1% were working or had worked on weight control; 4. Medical students had better knowledge about obesity than others; 5. Information sources: book/newspaper/magazine 48.9%, TV/internet/radio 33.7%; 6. Weight control motivations: improve appearance/confidence, wearing fashionable clothes; 7. Causes of obesity, 48% chose unhealthy diet, 43% chose “lack of exercise”, 6.8% chose “gene”; 8. Students from rural areas had less knowledge about obesity than urban students.
Mo et al. 2020^52^	Qual	Convenience sampling, In‐depth interview, instrument‐measured BMI; interpretive content analysis.	N = 50 (all F), all pregnant women; age range: 22–40 y, mean age 31 y; weight status prior to pregnancy, 26% were living with overweight, 6% were affected by obesity, 62% healthy weight, 6% underweight; 60% lived with their parents.	18.5–24.9 healthy weight, >25 overweight, >30 obesity	1. Participants used pregnancy as an excuse for not doing exercise; 2. Pressure from family members to eat more; 3. Family members thought “the fatter the better”; 4. Some considered being overweight is okay; 5. Internet information was confusing and unreliable; 6. They did not understand the importance of weight control; 7. Family members (especially expectant grandparents) greatly influenced pregnant women's lifestyles through overprotection, traditional and conservative ideas and practices; 8. A lack of reliable knowledge or guidance on gestational weight control. 9. They believed that “pregnancy is a time to eat for two and rest”; “physical activity can harm the foetus”; and “gentle exercise (e.g., walking or stretching) is sufficient”.
Peng et al 2014^53^	Quant Cross‐sectional study	Cluster random sampling; used a published questionnaire; instrument measured BMI.	N = 735 (M: n = 369, F: n = 366), university students, age range 18–25 y, mean 20.9 ± 1.42 y; male mean BMI 21.13 kg/m^2^, female mean BMI 19.4 kg/m^2^; female underweight 33.9%, male underweight 13.8%.	no information	1. All participants believed that obesity has negative impact on social life; 2. 70.75% thought people with overweight and obesity have more psychological problems than people with health weight; 3. 7.76% students knew how to calculate BMI; 4. 67.35% student did not want their future partner to be with overweight; 5. More female than male students actively looked for knowledge to control weight; 6. 95.37% would not use weight loss medicine. 7. Information source: TV/broadcast 58.8%, book/newspaper/magazine 58.5%, internet 58.0%.
Shujin Lian 2012^54^	Quant Cross‐sectional study	No information about sampling method; author adapted four published questionnaires, all were validated; self‐reported BMI.	N = 298 (all F), university students, age range: 19–24 y, mean age 21.1 ± 1.4 y； Mean BMI 19.9 ± 2.4 kg/m^2^； mean TV watching time 14.7 ± 11.7 hours per week.	18.5–23.9 healthy weight, 24–27.9 overweight, ≥28 obese	1. The higher the respondents' BMI, total television viewing time, and internalizations of societal ideals, the higher their reported body dissatisfaction and drive for thinness; 2. The higher the respondent's self‐esteem, the lower their reported body dissatisfaction and drive for thinness.
Sun & Su 2005^55^	Quant Cross‐sectional study	Stratified cluster random sampling in a university; self‐developed questionnaire; self‐reported BMI.	N = 746 (All F), age range: 17–24 y, university students; mean age: 20.3 ± 1.7 y; BMI range: 14.2–28.4 kg/m^2^, underweight: 30.7%, healthy weight.: 61.8%, Overweight: 4.7%, 25–29.9 kg/m^2^, obese: 2.8%.	≤18.5 underweight, 18.5–22.9 healthy weight, 23–24.9 overweight, 25–29.9 obese	1. Participants in the group with obesity were unhappy with their weight; 2. All participants preferred slim body figure; 3. Weight loss motivations: increase confidence 52.9%, pursuing beauty 51.5%, for health 43.3%; 4. Information sources: books and magazines 59.7%, friends 54.25; 5. 80% have thought about weight loss; 6. Weight loss methods: 32.6% exercise, 25.3% dieting.
Tanenbaum et al 2016^56^	Quant Cross‐sectional study	Secondary study; used data from a survey conducted in 2003–2004; multiple‐stage random sampling; piloted questionnaire which was adapted from the US youth risk behavior surveillance system; instrument‐measured BMI.	N = 902 (all F), university students; age range: 18–25, mean age 20.8 ± 1.4; all resided in urban areas; 10.5% overweight, 6.2% obese.	healthy weight 18.5–22.9, overweight 23–24.9, obese ≥25	1. 50.8% perceived themselves as either relatively heavy or too heavy; 2. 46% misperceived their weight, among which, 35.1% inaccurately considered themselves as overweight; 3. 48.2% were attempting to lose weight, 33.1% were aiming to maintain current weight; 4. 20.2% reported trying extreme methods such as fasting, laxatives, purging, smoking to control weight; 5. Only 21.5% reported using a combination of exercise and a reduced fat and calorie diet to control weight; 6. Attempts to control weight were related to actual and perceived weight status, but not to increasing exercise or fruit/vegetable intake.
Tao & Zhong 2010^57^	Quant Cross‐sectional study	Convenience sampling (asked pupils in two schools to give the questionnaire to their mothers); self‐reported BMI; used the eating disorder inventory −2 which was validated.	N = 236 (all F), pupils' mothers; age range 30–59 y, 30–39 y group 59.3%, 40–49 y group 38.1%, 50–59 y group 2.6%; mean BMI 21.49 ± 2.98 kg/m^2^;	very underweight ≤17.5, underweight 17.5–19, healthy weight 19–24, overweight 24–30, obesity ≥30.	1. 78% healthy weight females showed dissatisfaction with their weight and wanted to reduce it. 2. The 50–59 y age group had the higher mean BMI than 30–39 y and 40–49 y group; 3. The younger group (30–39 y) and middle age group (40–49 y) expressed the desire to lose weight at a lower BMI, compared to the older group (50–59 y); 4. People with overweight and obesity were unhappy with their weight; 5. The 30–39 y and 40–49 y age group females with BMI = 19 had the lowest dissatisfaction score; 6. T50–59 y age group females with a BMI = 24 had the lowest dissatisfaction score.
Tsou &Tsou 2003^58^	Quant Cross‐sectional study	Convenience sampling, self‐developed questionnaire; instrument‐measured BMI;	N = 1367, (M: n = 449, F: n = 918), residents who did free health check in a hospital during July‐Dec 2007; age: over 40 y, 40–49y 45%, mean age 53 y; mean BMI 23.6 kg/m^2^; obese 16%.	overweight 24.2–26.4, obesity >26.4	1. 42% of participants misjudged their weight status. 28% females within the healthy weight range considered themselves as overweight; 2. Participants with self‐identified overweight had greater nutrition knowledge than others; 3. Education and age had an impact on obesity in females but not males; 4. Health knowledge was inversely related to the probability of being affected by obesity; 5. Elderly females appeared to carry more excess weight; 6. White‐collar male workers were less likely to be living with obesity.
Wenjie Ling 2007^59^	Quant Cross‐sectional study	Randomized simple sampling process; using questionnaire, some interviews	N = 600 (F: n = 248, M: n = 352), university students; all were living with obesity; no other demographic information.	no information	1 35.2% of participants did not think that obesity had an impact on their life. Over half considered obesity influenced their lives significantly; 2. 87.3% of students with obesity misjudged their weight status; 3. 84.7% wanted to lose weight. 20.3% were actively attempting to lose weight; 4. Those who gave up on weight loss attempts stated they either lacked confidence or were scared of weight‐loss side effects; 5. 77.5% had tried dieting; 73.9% tried exercise; 10.6% tried acupuncture. 6. 80.1% of students were not happy with their weight loss results; 7. Many had tried extreme weight loss methods.
Wong & Huang 1999^60^	Quant Cross‐sectional study	Multiple‐stage random sampling; self‐developed and piloted questionnaire; instrument‐measured BMI.	N = 1057 (all F), university students, mean age 20.54 ± 2.21y; mean BMI 20.0 ± 2.0, mean desired BMI 19.0 ± 1.2；severely underweight 3,3%, underweight 18.9%, acceptable weight 61.5%, overweight 13.6%, obese 2.6%.	BMI < 16.9 severely underweight, 16.9–18.5 underweight, 18.5–21.7 healthy weight, 21.7–25.1 overweight, >25.1 obesity	1. More than 20% of subjects in both the severely underweight and the underweight categories and more than 50% of subjects in the acceptable‐weight category indicated that they wanted to lose weight; 2. Only 16.2% of subjects were classified as overweight or obese, yet 51.4% of the subjects perceived themselves as either overweight or obese; 3. The dissatisfied dieting group tended to measure their body weight more frequently, they also spent more time exercising and reading nutrition information; 4. Dieters were often preoccupied with food and calorie counting and had a great interest in reading literature about food and body weight; 5. Skipping lunch was a typical weight control method for dieters.
Worsley et al. 2017^61^	Quant Cross‐sectional study	Cross‐national study; convenience sampling in a volunteer pool ran by online market research company; self‐developed questionnaire; self‐reported BMI.	N = 807 (F: n = 461, M: n = 346), household food providers living in Shanghai; age range 18–64 y, mean age: 37.8 ± 10.5 y；mean BMI 23.6 ± 6.9 kg/m^2^; 77% were married.	No info	1. Weight concern: 10.5% very concerned, 38.7% somewhat concerned, 28% a little concerned. 2. 42.5% had tried to lose weight; 3. 55% had previously heard of the BMI; 4. Obesity causes: over 80% of people thought eating oversized servings of foods, regular consumption of fast foods, and overconsumption of sugar‐sweetened drinks caused obesity; 5. 67.9% of people were not aware of the health risks associated with obesity; 6. 65.4% of people chose “lack of availability of healthy foods” as a contributor to obesity, 87% chose “lack of physical activity opportunities”, 61.8% chose “genes”, 62.3% chose “modern technology”, 64.9% chose “the promotion of unhealthy foods, 41.3% chose “the low cost of unhealthy food”; 7. Weight control methods: 87.5% chose “establishing an exercise routine”, 68.9% chose “not sitting down for longer than 20 min”, 67.9% chose “not eating sweetened foods”; 8. Fewer Shanghainese people considered environmental influences to have a significant impact on obesity than respondents in Melbourne.
Wu et al. 2022^62^	Quant Cross‐sectional study	Exploratory study; convenience sampling, instrument‐measured BMI; self‐developed questionnaire.	N = 37,492 (M: n = 18,249, F: n = 19,243), adult residents lived in Chongqing city and went for health‐check in a hospital, no metabolic disease; mean age 42 ± 11.7 y; 1.8% were medically diagnosed as obese.	≥27.5 obesity	1. Only 2.1% of participants thought their close relatives were living with obesity. 2. Adults who perceived their close relatives as obese were more likely to engage in weight control behaviors; 3. Individuals with obesity had higher weight control behavior scores than those without obesity. 4. Those who perceived their close relatives as living with obesity had higher weight control behavior scores compared to those who did not; 4. People with obesity were more likely to think their relatives were also affected by obesity.
Zhang et al. 2022^63^	Quant Cross‐sectional study	Convenience sampling (within researcher informal network) and snowball sampling; self‐reported BMI; self‐developed and piloted questionnaire.	N = 184 (F: n = 70, M: n = 114), age range: 18–45 y; 19–35 y age group: 94.5%; self‐reported BMI; 102/184 were rural–urban migrants; 28.5% rural–urban migrants were with overweight or obesity; total sample underweight 15.8%, healthy weight 57.6%, overweight 19.6%; obesity 7.1%; 26–35 y: 100 (54.3%) 36–45 y: 10 (5.5%) 15.8% underweight., 57.6%: healthy weight., 19.6% overweight 7.1%: obese. Average BMI 22.1 kg/m^2^. 29 were living with overweight or obesity.	18.5–23.9 healthy weight, 24–27.9 overweight, ≥28 obesity	1. Most migrants (75%) agreed that obesity is a disease; 2. 93% migrants believed that obesity seriously threatens health; 3. 87.3% migrants vs 54.5% rural residents believed that obesity is due to a lack of willpower; 4. 69.6% believed that obesity is related to bad lifestyle habits and food addiction (58.7%); 5. Over half of both groups believed that obesity is a biological factor caused by genetic and hormonal imbalances and slow metabolism; 6. Majority of migrants and rural residents agreed that curing obesity can reduce risks of other diseases; 7. 69% believed weight loss is personal responsibility; 8. Low acceptance of pharmacotherapy and bariatric surgery; 9. 78.8% believed that pharmacotherapy was dangerous, while 25% thought it was effective for weight loss.
Xiang et al 2020^64^	Quant Cross‐sectional study	Multi‐stage stratified random sampling; Used questionnaire developed by Sichuan CDC; justified sample size; self‐reported BMI.	N = 487 (M: n = 251, F: n = 236), residents in Tongjiang county; age range: 15–81 y, 23.6% in 45–55 y age group; 60.2% self‐reported themselves as healthy	no information	1. 90.4% knew that obesity increases the risk of cardiovascular diseases; 2. 65.5% had basic knowledge about obesity; 3. The higher the education level the participant had, the better knowledge about obesity.
Xingzhu Shen 2010^65^	Quant Cross‐sectional study	Stratified cluster sampling in one university; self‐developed questionnaire; self‐reported BMI.	N = 1166 (All F), university students. No other information provided.	no information	1. 27.5% of students considered obesity as a sign of health, 34.4% considered obesity as a disease; 2. 43.8%% believed obesity had little or no negative impact on health; 3. 58.7% students who were actively trying to control their weight were within the healthy weight range; 4. Weight control methods: exercise 60.3%, diet 31.8%.
Yao et al 2015^66^	Quant Cross‐sectional study	Stratified cluster random sampling in a university; self‐development questionnaire, self‐reported BMI.	N = 283 (all F) university students; age range: 17–25, mean age 20.3 ± 1.7y; healthy weight: 66.8%, overweight and underweight: 2.8%, obesity: 0%. Mean BMI 19.5 ± 2.0; mean expecting BMI: 17.4 ± 1.4	18.5–23.9 healthy weight, 24–27.9 overweight, ≥28 obesity	1. 66.8% were within the healthy weight range, but 81.6% wanted to achieve an underweight BMI; 2. 40.6% considered themselves to be obese but only 2.7% were actually living with obesity; 3. 39.2% believed obesity only affects appearance, 7.4% believed obesity has no impact on health; 4. 81.3% had weight loss intentions; 5. 78.1% students' motivation was to improve confidence and appearance; 6. Information sources: TV, internet, magazine; 7. Weight loss methods: 60.3% adjusted diet pattern, 19.2% dieting, 4.6% exercise; 8. 71.5% considered the reason for failed weight loss attempts was due to their inability to persist with the weight loss methods in the long run.
Yin et al 2017^67^	Quant Cross‐sectional study	Stratified sampling in one university; no information about the questionnaire development; Self‐reported BMI.	N = 415 (M: n = 224, F: n = 191); university students from city or rural background; 10.4% overweight, 1% obese.	18.5–23.9 healthy weight, 24–27.9 overweight, ≥28 obesity	1. Over half of the female university students believed that they needed to lose weight; 2. 39.5% of male students from city background and 16.8% from rural areas believed that they needed to lose weight; 3. 77.6% students with healthy weight were working on reducing weight; 4. 42.2% dieters' motivation was to improve confidence and appearance; 5. Female students chose dieting and aerobic exercise, male students preferred exercise rather than dieting.
Yunfei Lin 2009^68^	Quant Cross‐sectional study	No sampling information, instrument measured BMI; self‐developed questionnaire;	N = 1597 (no information about gender proportion), university students in one university; female mean BMI 21.1 kg/m^2^, overweight 4.0%, obese 3.6%, healthy weight 50.2%. Male mean BMI 21.2 kg/m^2^, overweight 6.5%, obese 13.5%, healthy weight 41.9%.	18.5–23.9 healthy weight, 24–27.9 overweight, ≥28 obesity	1. Female students considered thinness as ideal; 2. Male students living with overweight and obesity wanted to lose weight, however, male students within the healthy weight range wanted to increase weight; 3. 86% females were worried about weight, 47.4% males had concern about weight; 4. 46.8% females chose dieting and 57.7% males chose exercise for weight control.
Zhang & Chen 2008^69^	Qual	Pilot study, in‐depth interview, convenience sampling	N = 10 (F: n = 8, M: n = 2), people who attended the TCM clinic in a hospital during 1994; Age range: 25–65 y.	no information	1. Generally, participants thought TCM has its value for weight loss, such as no side effects, no need to do exercise; 2. The main concern for TCM was that it takes long time to see the effect.
. Zhang & Chen 2010^70^	Quant Cross‐sectional study	Convenience sampling, used multiple validated questionnaires (Cronbach's alpha 0.8–0.9).	N = 590 (M: n = 403, F: n = 187), All were educated people; age range: 23–69 y, mean age 40.4 y; self‐assessed weight status.	no information	1 33.2% thought they were living with overweight or obesity. 2. People with overweight and obesity did less frequent, short time exercise, compared to participants with healthy weight; 3. Participants with obesity had higher motivation for exercise than the ones with healthy weight; 4. Appearance and health were main motivations for participants with obesity; health and entertainment were main exercise motivations for participants with overweight and healthy weight.
Zhang et al 2007^71^	Quant Cross‐sectional study	Convenience sampling, self‐developed self‐validated questionnaire; instrument measured BMI.	N = 2264, (M: n = 1353, F: n = 911), government officers; age range: 20–82 y; mean age: 50.3 y; overweight: 24.0%, obese: 3.5%.	≥24 overweight, ≥ 28 obesity	1. Only 23.34% participants knew BMI classification; 2. 46.38% assessed obesity by comparing with other people; 3. The main information sources: TV & radio (62.17%), followed by book & magazine (45.26%); 4. 80.05% wanted to know how to prevent obesity; 5. They preferred to obtain obesity‐related knowledge from professionals and TV programs.
Zhang et al 2008^72^	Quant Cross‐sectional study	Stratified cluster random sampling, self‐developed questionnaire; instrument measured BMI.	N = 1614, all F, university students, age range: 17–25 y, overweight:13.9%, obesity:1.9%, healthy weight 69.6%.	18.5–23.9 healthy weight, 24–27.9 overweight, ≥28 obesity	1. Female university students tended to consider that they were overweight, despite their actual weight being within the healthy range. 2. Nearly 95% considered thinness as beauty; 3. The primary motivation for weight control was to pursue better appearance; 4. 62% wanted to lose weight; 5. Majority knew the basic knowledge of obesity; 6. The main weight loss method was dieting, followed by exercise; 7. 73.2% did not know BMI classification; 8. 67.0% believed obesity is influenced by gene.
Zhang et al 2016^73^	Quant Cross‐sectional study	Cluster sampling in Wuhan city; sample size justified; self‐developed questionnaire; instrument measured BMI.	N = 1395 (All M), occupational workers; including middle school students 369, civil servants 348, enterprise managers 320, organizational workers 322; age range: 18–55 y; underweight 5.7%, healthy weight 54.5%, overweight 32.5%, obese 7.4%; master's degree and above 57%.	18.5–23.9 healthy weight, 24–27.9 overweight, ≥28 obesity	1. The 51–55 y age group had the highest knowledge about obesity; 2. 90.6% knew that people with obesity should control their weight; 3. 27.7% understood the reasons of obesity; 4. Majority did not know how to calculate BMI.
Zhou et al 2006^74^	Quant Cross‐sectional study	Two‐stage random sampling method; questionnaire was developed by a governmental organization; instrument measured BMI.	N = 494 (M: n = 148, F: n = 346), <40 y: 14.6%, 41–50 y: 20.2%, 46.2% 51–60 y: 19.0%, 46.2% ≥ 61 y; overweight rate: 38%, Obesity rate: 62%; Male central obesity rate: 97.3%, Female central obesity rate:96.5%.	18.5–23.9 healthy weight, 24–27.9 overweight, >28 obesity	1 The most common chronic disease the participants had was hypertension; 2. 66.2% did not think that they need to lose weight. 77.5% participants did not do anything for weight control; 3. 66.6% intended to use exercise for weight loss. For those who did not want to use exercise, reasons were disease, being not interested in sports, time restriction, tired from working; 4. 70% knew that obesity is a risk for developing chronic disease; 5. Fewer participants knew how much cooking oil and exercise is appropriate.
Yang et al 2012^75^	Quant Cross‐sectional study	Chosen all students with overweight and obesity from two universities in Zhaoqing city; no sampling information; self‐developed questionnaire; instrument‐measured BMI	N = 355 (F: n = 209, M: n = 146), university students, age range 18‐25y; all were living with overweight or obesity.	18.5–23.9 healthy weight, 24–27.9 overweight, ≥28 obesity	1. 27.9% could use BMI correctly; 2. 49.9% considered obesity as a disease; 3. 81.7% believed obesity relates to diet; 4. 62.5% believed obesity relates to gene; 5. 51.8% students used dieting method, 8.2% used exercise, 17.8% used medicine; 6. The reasons they did not choose exercise for weight control: lack of knowledge about exercise, no interest in sports, no place for sports, disliking hardship.
Yan et al 2011^24^	Quant Cross‐sectional study	Custer sampling, self‐developed questionnaire, instrument‐measured BMI;	N = 7039, (M: n = 3058, F: n = 3981), Conducted in two districts (one urban and the other rural) in both Beijing and Zhengjiang; age range: 35–60 y, mean age 47.3 ± 6.7 y； healthy weight 41.7%, overweight 39%, obese19.3%.	18.5–23.9 healthy weight, 24–27.9 overweight, ≥28 obesity	1. Information sources: mass media 74.7%, professionals 16.6%; family or friends 28.9%; 2. 80.1% knew the health risks of living with obesity; 3. 43.9% knew obesity increased the risk of developing diabetes 4. Less than 10% knew about the BMI; 5. 89.6% believed it is necessary to control oil intake for weight control; 6. 98% believed exercise is important for health; 7. Participants with obesity consumed more oil/fat than participants with overweight and healthy weight.

**Abbreviations**: BMI = Body Mass Index; F = female; M = Male; n or N = number; Qual = Qualitative; Quant = Quantitative; TCM = Traditional Chinese Medicine; WTP = Willingness to Pay;  y = years of age.

The total sample size was 83,688, with 47,289 female (56.5%) and 36,399 male (43.5%) participants. Study sample sizes ranged from 5 to 37,492. Participants were aged between 18–81 years. The overall sample was more likely to consist of younger and more highly educated people, with 24 studies including solely university students. Females were more often researched, with 17 out of 53 studies recruiting solely female participants and only one study involved solely male participants. The occupations of the participants varied from university students, governmental workers, and retirees, to company employees. Health status ranged from those who perceived themselves to be healthy to people with chronic diseases. Two studies were conducted involving pregnant women living with overweight or obesity.^41^, ^52^ Most participants were within the healthy weight range, with only 10 out of 53 studies involving a sample where 50% or more of the participants were living with overweight or obesity.

Most studies utilized BMI for defining overweight and obesity, with only five studies^37^, ^38^, ^44^, ^50^, ^74^ collecting additional information on waist circumference or waist‐hip rate alongside weight and height measurements. Among these five studies, the results pertaining to waist circumference or waist‐hip rate were either unreported or omitted from data analysis.^37^, ^38^, ^50^, ^74^ Eighteen studies employed instrument‐measured BMI, while the remainder relied on self‐reported methods or did not provide relevant information. Sixteen papers did not provide BMI threshold information. Among the remaining 37 papers, researchers employed 8 different sets of BMI thresholds for overweight and obesity classification. For example, the Chinese version was the most commonly used,^24^, ^30^, ^31^, ^34^, ^35^, ^37^, ^38^, ^50^, ^51^, ^54^, ^63^, ^66^, ^68^, ^71^, ^72^, ^73^, ^74^, ^75^ followed by the international version^25^, ^32^, ^36^, ^39^, ^41^, ^52^ and the Asian‐pacific version.^28^, ^55^, ^56^ The remaining nine papers used uncommon BMI thresholds such as BMI ranges of 21.7–25.1 kg/m^2^ or 24.2–26.4 kg/m^2^ or 24–30 kg/m^2^ or 23–24.9 kg/m^2^ for defining overweight.^23^, ^28^, ^33^, ^49^, ^57^, ^60^, ^62^

*(**Chinese version**: BMI<18*.5 kg/m^2^ as *underweight, 18.5–24* kg/m^2^ *as healthy weight range (HWR), 24–27.9* kg/m^2^ *as overweight, ≥28* kg/m^2^ *as obesity; **International version**: BMI<18.5* kg/m^2^ *as underweight, 18.5–25* kg/m^2^ *as HWR, 25–30* kg/m^2^ *as overweight, ≥30* kg/m^2^ *as obesity; **Asia‐Pacific version**: BMI<18.5* kg/m^2^ *as underweight, 18.5–23* kg/m^2^ *as HWR, 23–25* kg/m^2^ *as overweight, ≥25* kg/m^2^ *as obesity)*.^26^, ^57^, ^60^

### 3.1 Direct and indirect methods to explore perception

The included studies used either direct or indirect methods to explore participants' perceptions of obesity. The direct method involved researchers directly asking participants' opinions, attitudes, or motivations, either via questionnaires, in‐depth interviews, or focus groups. The majority of studies ((41 out of 53) utilized the direct method. Common topics in these studies included participants' self‐evaluated body weight status, satisfaction with body weight, perceived health risks of obesity, perceived causes of obesity, willingness or motivations for weight control, preferred weight control methods, and sources of obesity‐related information. Less common topics included the length of time spent living with overweight/obesity, emotional eating, desired body weight, and acceptance of weight loss drugs.

Conversely, 12 papers employed the indirect method to explore obesity perceptions, whereby researchers used a deductive approach to gather information from individuals. For example, perceptions were deduced from behavior analysis,^32^, ^36^, ^45^, ^46^, ^54^, ^57^ willingness to pay for weight loss therapies,^23^ motivation for exercise,^70^ and psychological status.^29^, ^31^, ^39^ Studies using indirect methods often utilized existing scales for data collection. For example, 9 studies employed a total of 14 validated scales, including the Attitude about Person with Obesity Scale,^28^, ^36^ Implicit Association Test,^29^, ^36^ Self‐Esteem Scale,^54^ The Eating Disorder Inventory,^54^, ^57^ The Hospital Anxiety and Depression Scale,^28^ Perceived Weight Stigma Scale,^43^ the Sociocultural Attitudes toward Appearance scale,^54^ the Contour Drawing Rating Scale^46^, the Willingness of weight control,^46^ The Chinese version of the Dutch Eating Behavior Questionnaire,^32^ The Physical Activity Rating Scale‐3,^32^ Antifat Attitudes Questionnaire,^31^ Weight Bias Internalization Scale,^31^ and the Body Areas Satisfaction Scale.^43^

### 3.2 Qualitative and quantitative methods to explore perception

In the nine qualitative studies, six studies employed purposive sampling,^26^, ^27^, ^40^, ^41^, ^47^, ^48^ two utilized convenience sampling,^52^, ^69^ and one study lacked sampling information.^30^ Regarding the data collection methods, most studies employed in‐depth interviews,^26^, ^27^, ^30^, ^40^, ^47^, ^48^, ^52^, ^69^ with only one utilizing the focus group method.^41^ Overall, these studies explored participants' motivations, experiences with weight loss struggles, and the cultural, societal, and environmental factors regarding obesity. There were five consistent findings 1. slimness is overwhelmingly preferred which caused an inferiority feeling among participants with overweight/obesity; 2. weight management is a constant struggle for participants; 3. Others' comments has a major impact on participants; 4. the motivation for weight loss is to gain social acceptance, rather than address health concerns; 5. traditional dietary habits, social norms, and personal values played a significant role in participants' obesity‐related perceptions. It is worth noting that five out of the eight studies focused primarily on people within the healthy weight range. The remaining studies included participants with overweight or obesity. However, these studies were designed to investigate participants' perceptions toward specific weight loss methods. Only one qualitative research paper explored the lived experiences of Chinese adults with excess weight.^40^

Among the 44 quantitative papers, comprising 43 observational cross‐sectional studies (16 English and 27 Chinese papers) and one intervention study, over half (24/44) employed various types of random sampling, including multiple‐stage sampling,^23^, ^39^, ^49^, ^50^, ^56^, ^60^, ^64^, ^74^ stratified sampling,^33^, ^43^, ^45^, ^67^ cluster stratified sampling^24^, ^42^, ^51^, ^53^, ^55^, ^65^, ^66^, ^72^, ^73^ or simple sampling.^31^, ^32^, ^59^ Fourteen studies used convenience sampling^23^, ^25^, ^28^, ^34^, ^35^, ^36^, ^38^, ^46^, ^57^, ^58^, ^61^, ^63^, ^70^, ^71^ while five did not specify their sampling methods.^29^, ^37^, ^44^, ^54^, ^68^, ^75^ Most studies had a small to medium sample size and were local in scope, not intended for broad population generalization. In the 43 cross‐sectional studies, researchers provided obesity‐related predefined statements for participants to choose. The topics were homogenous and the statements were simple. The overall results indicated a low awareness of BMI, fair knowledge of basic obesity concepts, high motivation for weight loss, lack of knowledge and skills on weight loss methods, and low satisfaction with body weight. In addition, the sources of participant's obesity‐related information varied, with mass media (television, radio, internet, books, magazines) being the main sources of information (accounting for over 60%), followed by friends or family members (9%–39.8%) and medical and health professionals (16.6%–26%).

### 3.3 Quality assessment results

1. The quality of the nine qualitative studies was variable and generally suboptimal. (detailed results in Supplementary Information Table S3) None of the studies provided information regarding the researchers' positioning (Question 6 in CASP), which made it difficult to assess the extent of researchers' influence on the findings. With the exception of the “researchers' position” domain (which no articles satisfied), five studies were considered to be of fair quality.^27^, ^40^, ^41^, ^47^, ^48^ The next most problematic domain was the ethical considerations (Question 7), which were unclear in three studies.^30^, ^52^, ^69^ One pilot study was deemed to be of low quality, due to its lack of rigorous data analysis and unclear presentation of findings.^69^ However, this low‐quality study made only a minimal contribution to the final thematic synthesis, as it focused on an uncommon topic (participants' experience and understanding of Traditional Chinese Medicine methods for weight loss).
2. The quality of the 43 cross‐sectional studies (16 English papers and 27 Chinese papers) ranged from poor to moderate based on their reported information (detailed results provided in Supplementary Information Table S4). Most studies published between 2019 and 2024^32^, ^35^, ^39^, ^42^, ^43^, ^62^, ^63^, ^64^ exhibited higher rigor compared to those published in earlier years, since these recent studies has less missing information (5–7 “No” or “Don’t know”s), compared to those published prior to 2019 which contained 7–15 “No” or “Don’t know”s. Additionally, studies published in English demonstrated better quality than those written in Chinese. The majority of studies (36/43) did not provide justification for their sample size. Sampling procedures were generally problematic, with 37 out of 43 studies either lacking clear recruitment details or providing no information about non‐responders. Seventeen studies utilized self‐developed questionnaires that had not been trialed, piloted, validated, or published previously. The reporting style of the methods and data analysis sections was generally poor, with nearly half (19/43) of the studies offering vague descriptions that hindered reproducibility. Due to a lack of details provided on data analysis methods by 15 studies, it was unclear if the results presented by these papers aligned with the analysis methods they described. Over half of the studies (24/43) did not discuss their limitations. Concerns were also raised regarding conflicts of interest declarations and ethnical approval, as 20 out of 43 studies did not provide funding information and only 14 out of 43 mentioned ethical approval.
3. The singular intervention study^29^ was assessed via the Cochrane RoB 2 tool. The risk of bias in this study indicated some concerns overall, due to the lack of information provided about the randomization process used and the potential selection of the reported result.

### 3.4 Thematic synthesis findings

Given that most of the 43 cross‐sectional studies were homogeneous in their study design, the individual findings gain validity as multiple studies found similar results, despite most using convenience small‐scale samples. Moreover, the findings from qualitative studies triangulate to the results from quantitative studies when investigating similar topics, such as motivation to lose weight, perceived method for weight loss. Each theme was supported by consistent findings in multiple studies. Therefore, the three thematic synthesis findings are considered to be of high certainty.

a Chinese adults connected obesity with appearance more than with health;

The majority of participants involved in the included studies were within the HWR and included a high proportion of young female adults. The overall results indicated a high intention to lose weight and considerable dissatisfaction with current body weight. Eighteen cross‐sectional studies found that 42.5% to 81.3% of participants, particularly females, intended to lose weight. Also, 11 studies identified the primary motivations for weight loss as improving self‐confidence and enhancing appearance, followed by health concerns^26^, ^30^, ^35^, ^38^, ^44^, ^50^, ^51^, ^55^, ^66^, ^67^, ^70^ This situation corresponded with the phenomenon that up to one‐third (7.42%–34.1%) of participants in earlier studies did not consider obesity as a risk for health,^33^, ^37^, ^59^, ^66^ but in contrast, the majority of participants believed that living with obesity could impair physical attractiveness and social life quality. Fifteen quantitative studies examined body weight satisfaction, revealing that 28% to 82% of participants were unhappy with their body weight.^28^, ^32^, ^33^, ^38^, ^44^, ^54^, ^56^, ^57^, ^59^, ^60^, ^61^ This dissatisfaction was partly attributed to inaccurate self‐evaluation of weight status, with 28.3% to 87.3% of participants misjudging their weight status.^28^, ^34^, ^35^, ^38^, ^42^, ^45^, ^50^, ^56^, ^59^, ^66^, ^67^ The most common misperception was among HWR participants who viewed themselves to be living with overweight or obesity. Three cross‐sectional studies also reported that a small proportion (10.39%–28.3%) of individuals with overweight perceived themselves to be at a healthy weight.^38^, ^50^, ^59^ People with high BMI were generally aware of their excess body fat. Interestingly, compared to females, males adopted a more relaxed attitude toward body weight, as less males misperceived their body weight status.^23^, ^43^, ^68^ Only one study suggested that male university students showed stronger negative attitudes toward obesity than females.^31^ However this study was considered as low‐quality as it provided limited information on data collection and analysis.

The reason for weight misperceptions may have been related to the poor awareness of BMI that many study participants had. Nine studies found that 70–90% of participants either did not know what BMI was or did not know how to use it correctly.^24^, ^38^, ^50^, ^53^, ^61^, ^71^, ^72^, ^73^, ^75^ In addition, six cross‐sectional studies consistently indicated that participants' self‐evaluation of weight status rather than actual weight status was a more reliable predictor of their weight management behaviors.^23^, ^43^, ^45^, ^46^, ^56^, ^60^

The observed “misperception” of weight status was also closely tied to the differing evaluation standards used by researchers and participants. Researchers generally used BMI to classify overweight and obesity, whereas participants often used non‐clinical, often external, judgments such as clothing size, comparisons with others, using weight scales, or comments from others.^26^, ^30^, ^37^, ^42^, ^65^, ^71^ Two studies explicitly clarified that participants acknowledged their “overweight” status was based on strict beauty standards rather than health risk.^26^, ^30^ The appearance standard partly explained the gender difference regarding the attitude toward obesity. For example, females perceived that society imposed a stricter body weight standard on themselves, thus they had a stronger motivation to lose weight than males.^23^, ^30^, ^37^, ^40^, ^43^, ^49^, ^68^ The appearance standard is also linked to the preference for slimness among female participants. Several studies found that a slim figure was highly desirable among young females,^26^, ^30^, ^40^, ^55^, ^57^, ^66^, ^68^, ^72^ Two studies indicated a preference for a BMI of around 19 kg/m^2^, bordering on the underweight category for Chinese.^57^, ^66^ Studies also show that females preferred diet control whereas males preferred exercise for weight control.^30^, ^49^, ^67^, ^68^

b Chinese adults lacked practical knowledge to manage obesity

Study participants appeared to have a reasonable understanding of basic obesity knowledge (referring to the basic literacy about obesity, e.g., health risks, causes of obesity), yet they lacked practical knowledge on how to address obesity. Seventeen studies assessed the participants' awareness of the health risks associated with obesity.^24^, ^33^, ^35^, ^37^, ^38^, ^42^, ^44^, ^50^, ^52^, ^53^, ^61^, ^63^, ^64^, ^65^, ^66^, ^73^, ^74^, ^75^ Studies showed an increasing trend in the awareness of obesity‐related health consequences. For example, four cross‐sectional studies focused on young‐to‐middle‐aged adults living in Mainland China and used self‐developed questionnaires,^63^, ^65^, ^75^ finding that 34.4% of participants in a 2010 study,^65^ 49.9% of participants in a 2012 study,^75^ 75% of participants in a 2022 study,^63^ and 76.8% of participants in a 2024 study,^35^ considered obesity as a chronic disease. However, there were significant gaps in practical weight management knowledge. This is evident through four key points: Firstly, the low BMI awareness indicated participants' lack of knowledge in assessing their weight status. Secondly, unrealistic weight loss goals or problematic methods for weight loss (e.g., losing 6 kg per month, long‐term use of anti‐obesity drugs, losing weight quickly without effort, aiming to lose up to 20% of their weight in contrast to a doctors' recommendation of 12%, no exercise, fasting, no endeavor to change fruit and vegetable intake, purging, smoking) were frequently reported, especially among those with obesity.^30^, ^33^, ^35^, ^44^, ^49^, ^56^, ^74^, ^75^ Thirdly, Chinese adults tended to rely on self‐resourced obesity‐related knowledge rather than approaching healthcare professionals. Only 16.6%–26% of participants sought information from health professionals.^24^, ^33^, ^50^, ^55^, ^71^ Fourthly, participants commonly viewed overweight and obesity as personal responsibilities, emphasizing exercise and “diet control” as the weight loss methods. The ambiguity of “diet control” methods further complicates the issue. The term was used inconsistently across studies: 10 studies equated it with dieting, while four referred to dietary adjustments, and 16 studies did not explain the term. In summary, while participants may be aware of obesity's risks, their practical knowledge on how to address obesity was largely inadequate.

c Dealing with obesity is perceived as a solitary journey

Dealing with obesity was reported to be an emotionally taxing and isolating journey for Chinese adults.^26^, ^40^, ^41^ A predominantly negative attitude toward obesity prevailed, e.g., “people with excess weight are clumsy, lazy and unhealthy, physically unattractive”^34^, ” people with obesity have more psychological problems”,^53^ “the reason for obesity is that people lack of willpower”.^63^ Seven studies found that 62–100% participants expressed implicit or explicit negative attitude toward obesity.^28^, ^29^, ^30^, ^31^, ^34^, ^36^, ^53^ Three quantitative studies revealed that commenting on others' body weight was common in everyday life.^26^, ^30^, ^40^ Internalized obesity self‐blame was also reported in five studies, where participants internalized these negative beliefs such as “feeling sad and inferior, feeling rejected”^26^ “feeling shameful and embarrassed, fat is ugly”^30^ “feeling profoundly hurt”^40^ “psychological distress, depression, anxiety”^28^ “feeling like a failure”.^41^ The negative attitude toward obesity seems stem from participants' subjective understanding of its causes. For instance, 12 studies explored participants' views on the causes of obesity, with “lack of exercise” and “overeating” being the most frequently reported reasons,^37^, ^38^, ^51^, ^61^, ^75^ followed by genetic reasons.^37^, ^38^, ^51^, ^61^, ^63^ Lack of willpower^63^, ^66^ and irregular meals^38^, ^50^, ^71^ were also occasionally mentioned. These responses indicate a blaming mindset that largely attributes obesity to personal lifestyle choices. The combination of a social environment, internalized accusation, and the mindset of considering obesity being a self‐responsibility together led to self‐hatred, lack of confidence, and self‐devastated feelings in many participants who considered themselves as living with overweight or obesity. This is evident in several studies where participants describe their weight loss experience as miserable, lonely, and unsupported.^26^, ^28^, ^30^, ^40^, ^41^, ^43^ Taken together, these findings underscored the emotional toll and social challenges faced by individuals dealing with obesity in a context where the negative attitude toward obesity is pervasive and often internalized.

## 4 DISCUSSION

To our knowledge, this is the first exhaustive systematic review exploring extant literature regarding Chinese adults' perceptions toward overweight and obesity. It included all types of study designs and Chinese adults residing both within and beyond China. It is a bilingual review incorporating English, simplified, and traditional Chinese written papers in order to mitigate potential publication bias. This review found that *(i)* Chinese adults tend to misperceive their weight status, largely stemming from limited awareness of BMI and societal norms idealizing thinness, leading to high dissatisfaction with body weight and high motivation to pursue weight loss. Females perceived a stricter weight ideal than males; (ii) Despite a pronounced motivation for weight management, there is a lack of practical knowledge regarding sustainable strategies. Problematic methods and unproductive perceptions were prevalent among this population; *(iii)* Chinese adults perceived dealing with obesity as a solitary journey, influenced by the pervasive negative societal attitude toward obesity and the prevailing mindset emphasizing personal responsibility for weight‐related issues.

### 4.1 Lack of practical knowledge on weight management

This review shows a growing awareness of obesity as a health risk and that Chinese adults who perceived themselves as with overweight or obesity are strongly motivated to lose weight, driven by both aesthetic and health considerations. Notably, even HWR females exhibited a desire for thinness. This may be potentially influenced by traditional beauty norms which encouraged females to be slim, fragile, and petite throughout most dynasties (except for the Tang dynasty).^76^ It may also be influenced by the contemporary mass media and popular culture propagating the thin ideal.^77^ In contrast, Chinese males appeared to demonstrate a more realistic self‐assessment of their current body weight than females. Males within HWR very rarely expressed a desire to lose weight.^68^ Nevertheless, it is obvious that most Chinese adults with overweight/obesity who participated in these studies were highly motivated to lose weight. Interestingly, this contradicts findings from other non‐Chinese‐specific reviews which suggest a lack of motivation among individuals with obesity, particularly in those with more weight to lose at baseline, those with concomitant mental health concerns, and low health‐related quality of life.^78^, ^79^, ^80^ This review offers a nuanced perspective on the intertwined relationship between motivation (desire or willingness to achieve a goal)^81^ and self‐efficacy (subjective perception of capability to achieve a goal).^82^ It posits that low self‐efficacy may masquerade as low motivation when participants' perceptions are not adequately explored. Low self‐efficacy can engender a state of learned helplessness,^83^ dampening enthusiasm for behavior change, which may be misinterpreted as low motivation by external observers. Given that excess weight is not only linked with health risks but also appearance and quality of social life, coupled with a societal negative attitude toward obesity, the high motivation to lose weight among Chinese adults is nearly guaranteed, which has been demonstrated in this review that Chinese adults participating in the included studies had a high motivation to lose weight. We should be cautious to label individuals as having “low motivation” as this narrative implies personal culpability. Instead, working on addressing low self‐efficacy may shift the emphasis away from blaming individuals and may yield more productive outcomes.

Low self‐efficacy in weight management often arises from repeated and unsuccessful past experiences of weight loss and a lack of practical knowledge and skills. For example, most Chinese adults understand that they need to work on diet adjustment and exercise, however, not many people know how to develop a sustainable and practical lifestyle change. This involves knowledge and skills that seemed unfamiliar to many participants. This knowledge gap maybe attributed to various factors within Chinese culture. **Firstly**, the majority of individuals with excess body weight may live without significant health complications or physical limitations, which may diminish the urgency of seeking professional help. Also, in Chinese culture, weight issues are commonly perceived as an appearance issue rather than an urgent medical concern,^84^ thus, individuals tend to seek freely available information from mass media or acquaintances rather than paying for consultations with health professionals. **Secondly**, for the Chinese population, obesity is a relatively recent public health issue. Thus, there is a scarcity of experts who have mastery knowledge of obesity in the field. It is not uncommon to see healthcare professionals being less confident or lack of knowledge/training in dealing with overweight and obesity.^85^, ^86^, ^87^ Currently, there are more fragmented approaches to weight management. For example, allied health professionals (dietitians, exercise physiologists), fitness coaches, psychologists, and weight loss surgeons provide support and strategies to promote weight loss, but in isolation rather than working collaboratively. The information from different sources may be inconsistent and contribute to public confusion. **Thirdly**, the healthcare system underpinned by the medical model, such as hospitals, is not well‐equipped to address weight management issues on a population level, as weight loss requires sustained individual effort over time. An ideal solution for this public health issue should be cost‐effective, sustainable, and involve long‐term counseling and support from trained health professionals. **Lastly**, the proliferation of commercial weight loss advertisements in mass media often promotes unrealistic claims and may lead to confusion among the public. The high exposure to mass media and exaggerated appealing statements may overshadow the realistic and evidence‐based guidance provided by healthcare professionals.

### 4.2 Negative attitude toward obesity in Chinese people

This review underscores the prevalent negative attitude toward obesity within Chinese society, which often causes self‐accusation among those living with overweight and obesity. This phenomenon is close to the concept of “obesity stigma” in Western culture, which was translated as “肥胖污名化” by many Chinese researchers. Interesting, among the 53 included studies, only two studies explicitly used the term “obesity stigma” (the discrimination toward people based on their body weight and size).^28^, ^43^ Three studies used the term “negative attitude”.^29^, ^31^, ^34^ In total, only seven studies touched on this topic, indicating a relative lack of scholarly attention. Contrary to the Western context, where obesity stigma prevention has emerged as a prominent research focus due to its detrimental impact on weight management, the perspective of Chinese individuals toward “obesity stigma” may be different, due to the predominance of Confucianism ideologies and the long‐rooted Buddhist culture, which promotes self‐discipline and constructive responses to criticism.^88^, ^89^ Consequently, Chinese individuals may harbor a distinct cognitive framework when confronted with criticism and negative feedback. This cultural disposition is reflected by many folk proverbs educating Chinese people to view criticisms or adversity positively, such as “良药苦口利于病, 忠言逆耳利于行 (good medicine tastes bitter but is beneficial for health promotion, loyal advice is harsh but beneficial for one's conduct)”, “吃得苦中苦, 方为人上人 (enduring hardship leads to greatness)”. Furthermore, Individuals living in collectivist societies often demonstrate a propensity to conform and adhere to other's judgment in order to harmonize with broader social expectations, prescribed roles, and standards.^90^ This resonates with findings from one of the included studies that Chinese females' pursuit of a slender physique is motivated by a desire for social approval.^26^ In contrast to Western cultural norms where negative judgments about obesity are regarded as a form of discrimination, Chinese individuals may perceive such comments as motivational stimuli or well‐intentioned persuasion for greater societal good rather than discriminatory acts. This may partly explain that Chinese adults living with overweight and obesity tried to solve the problem on their own, as shown in this review, and inevitably leading to a lonely journey. Further research endeavors should thus be directed toward elucidating this nuanced perspective.

Regardless of how the Chinese population views negative attitudes toward obesity, self‐blame among individuals perceiving themselves as with overweight or obesity is pervasive. This introspective condemnation may serve as a catalyst for some individuals to adopt weight management practices, while for others, it may precipitate mental health complications and adversely impact overall well‐being, leading to social withdrawal behaviors. For healthcare practitioners working with the Chinese demographic, it might be useful to openly discuss obesity stigma and direct individuals toward a constructive perception of obesity stigma, given they may encounter it on a daily basis. On the other hand, comprehensive societal education on obesity stigma and the implementation of prevention strategies are imperative. In addition, it is pertinent to recognize that excess body weight does not invariably correlate with health status, and holistic well‐being encompasses not only physical but also mental health considerations. When considering individuals' mental health, adherence to the principles espoused by the Weight Issues Network such as “respect the individual's right to choose their path” is judicious.^91^ Accordingly, for some individuals, this entails weight reduction to mitigate health risks, whereas others may remain content without pursuing weight reduction if there are no immediate health concerns.^91^

### 4.3 Methodology limitations and future directions

Over 80% of the included studies employed quantitative observational cross‐sectional surveys to explore perceptions of obesity among Chinese adults. Serving as a “snapshot” depiction of perception for certain groups, such surveys possess advantages, such as being low‐cost, easy to conduct, yielding quick results, and posing rare ethical challenges.^92^ These studies illustrated a range of perceptions regarding various aspects of obesity and the prevalence of particular perceptions among Chinese adults spanning from 1999 to 2024. This is useful for establishing preliminary evidence for future study.^92^ Given the inherent limitations of cross‐sectional studies, it is understandable that the perceptions probed via such studies may only scratch the surface. Obesity is a complicated issue involving interrelated and multifaceted perceptions. To delve into deeper layers of perception regarding obesity, a qualitative approach could offer a more comprehensive exploration of the phenomenon.^93^ However, less than 20% of included studies utilized qualitative methods, which indicates a major research gap. Furthermore, the overall sample consisted primarily of younger urban individuals within the HWR, with nearly 60% being females, and minimal representation from rural areas. Thus, the total sample fails to comprehensively represent the diversity in the general Chinese (华人) population, particularly those who are living with overweight and obesity. The findings of this review can only be applied to young‐middle‐aged Chinese female city dwellers living in Mainland China and Taiwan regions. It would be of great value if future studies target more male, more elderly, and more individuals with overweight or obesity, given their pronounced need for weight‐related interventions. Moreover, only one out of 53 studies involved some Chinese immigrants, yet the specific findings for this subgroup remain unclear due to inadequately reported details.^25^ Notably, a substantial number of Chinese individuals have migrated overseas, particularly to English‐speaking developed countries.^94^ The distinctive social, cultural, and environmental exposures in the Western context are likely to influence the perceptions toward obesity among Chinese immigrants. Hence, future research should allocate resources to Chinese immigrants regarding this topic.

People's perceptions are ever‐evolving. There was no nationally representative data at certain time points but only discrete small‐scale studies on different groups of people spaning over 20 years. This added complexity to synthesize the findings. Examination of these studies reveals several knowledge gaps in the field. Firstly, there is a dearth of research investigating Chinese immigrants residing in Western countries. Secondly, hearing from individuals who have weight issues is important. Yet, there is a lack of studies elucidating the lived experience of Chinese individuals with overweight and obesity. Accessing these individuals may pose challenges. However, this situation underscores the importance of working with them specifically, in order to untangle the physical and emotional barriers of weight management. Thirdly, obesity stigma might represent a concealed and understudied issue, also a unique concept within the Chinese context, warranting further exploration. Fourthly, human perceptions are profoundly influenced by life experiences, social norms, and information exposure. Regarding overweight and obesity, individuals' weight change history, current weight status, and exposure to mass media information may interchangeably influence perception. However, there remains a paucity of research in these domains.

## 5 CONCLUSION

This review identified three main themes regarding the perception of weight status and weight management among Chinese adults. Firstly, it was observed that Chinese adults often misperceive their weight status due to limited awareness of BMI and societal norms that idealize thinness, particularly impacting females who perceive a stricter weight standard compared to males. Secondly, despite a strong motivation for weight management, there is a notable lack of practical knowledge regarding sustainable strategies, leading to the prevalence of problematic methods and unproductive perceptions among this demographic. Lastly, dealing with obesity is perceived as a solitary journey by Chinese adults, influenced by the pervasive negative societal attitude toward obesity and the prevailing mindset emphasizing personal responsibility for weight‐related issues.

## CONFLICT OF INTEREST

The authors declare no conflicts of interest.

## Supporting information


**Table S1.** Database Search Strategy.
**Table S2.** Screening eligibility criteria.
**Table S3.** Quality assessment results for the nine qualitative studies via CASP.
**Table S4.** Quality assessment results for the 43 cross‐sectional studies via AXIS.
