# Supplementary material for: Exploring the perceptions of Chinese adults toward overweight and obesity: A systematic literature review
Source: Obes Rev. 2025 Feb 18;26(7):e13913. doi: 10.1111/obr.13913 (PMC12137042; doi:10.1111/obr.13913)
Supplement: Supplementary file 1 — Table S1. Database Search Strategy. Table S2. Screening eligibility criteria. Table S3. Quality assessment results for the nine qualitative studies via CASP. Table S4. Quality assessment results for the 43 cross‐sectional studies via AXIS. [file OBR-26-e13913-s001.docx]

**Supporting Information**

# **Exploring the perceptions of Chinese adults towards overweight and obesity: a systematic literature review**

[Yixi Wang-Chen](https://pubmed.ncbi.nlm.nih.gov/?term=Wang-Chen+Y&cauthor_id=36727422)^1^,  Hui Yang^2^, [Nicole J Kellow](https://pubmed.ncbi.nlm.nih.gov/?term=Kellow+NJ&cauthor_id=36727422)*^1^ & [Tammie S T Choi](https://pubmed.ncbi.nlm.nih.gov/?term=Choi+TST&cauthor_id=36727422)*^1^

1 Department of Nutrition, Dietetics & Food, Monash University, Melbourne, VIC, Australia.

2 Department of General Practice, Monash University, Melbourne, VIC, Australia.

*co-senior authors

**Correspondence:** Yixi Wang-Chen, Mobile: +61 4 1340 6056 Fax number: +61 3 9902 4278 Email: [Yixi.Wang@monash.edu](mailto:Yixi.Wang@monash.edu) Address: Department of Nutrition, Dietetics & Food, Monash University. 264 Ferntree Gully Road, Notting Hill VIC 3168 Australia

| **Table S2: Screening eligibility criteria** | | |
| --- | --- | --- |
|  | Include | Exclude |
| **Sample** | Chinese adults (≥18 years) living in China and other countries, with any health condition. | Not Chinese, Children and Adolescent, Second generation Chinese people |
| **Phenomenon of interest** | Perception towards overweight or obesity | **1** Underweight, normal weight, eating disorder, body image study that is not weight-related.  **2** Studies focused on body image or weight control, and did not mention overweight/obesity in its abstract.  **3** Studies focus on weight estimation but no participant's perceptions on overweight/obesity. |
| **Study design** | Any research design, e.g., survey, interview, intervention, longitudinal research method |  |
| **Evaluation** | 1 Any Chinese adults' perceptions, beliefs, attitudes, knowledge toward obesity and its management.  2 Chinese adults' perception of their own weight. | **1** Parental opinion for their children's weight, medical practitioners’ opinion towards patients living with overweight or obesity.  **2** Survey about hypertension, cardiovascular disease, or other chronic diseases, overweight/obesity knowledge/attitude was mentioned once and is not the focus of the study.  **3** Health or nutrition knowledge survey, if it does not mention overweight/obesity in its abstract. |
| **Research type** | All types of original study, Including qualitative, quantitative, or mixed method studies | Review, conference paper |

| **Table S3: Quality assessment results for the nine qualitative studies via CASP** | | | | | | | | | | |
| --- | --- | --- | --- | --- | --- | --- | --- | --- | --- | --- |
| **Author** | **1 Was there a clear statement of the aims of the research? (If you have comments, put into the "other" option. this function applies to all questions)** | **2 Is a qualitative methodology appropriate?** | **3 Was the research design appropriate to address the aims of the research?** | **4 Was the recruitment strategy appropriate to the aims of the research?** | **5 Was the data collected in a way that addressed the research issue?** | **6. Has the relationship between researcher and participants been adequately considered?** | **7. Have ethical issues been taken into consideration?** | **8. Was the data analysis sufficiently rigorous?** | **9 Is there a clear statement of findings?** | **10 How valuable is the research** |
| Chang et al 2004 ^1^ | Yes | Yes | Yes | Yes | Yes | No | Yes | Yes | Yes | Yes |
| Chang et al. 2021 ^2^ | Yes | Yes | Yes | Yes | Yes | No | Yes | Yes | Yes | Yes |
| Chu, Yu-Wei 2008 ^3^ | Yes | Yes | Yes | Can't tell | Yes | No | Can't tell | Yes | Yes | Yes |
| Keyser-Verreault 2022^4^ | Yes | Yes | Yes | Can’t tell | Yes | No | Yes | Yes | Yes | Yes |
| Lee et al. 2023 ^5^ | Yes | Yes | Yes | Yes | Yes | No | Yes | Yes | Yes | Yes |
| Liou & Bauer 2010 ^6^ | Yes | Yes | Yes | Yes | Yes | No | Yes | Yes | Yes | Yes |
| Liou et al 2007 ^7^ | Yes | Yes | Yes | Yes | Yes | No | Yes | Yes | No | Yes |
| Mo et al. 2020 ^8^ | Yes | Yes | Yes | Yes | Yes | No | Can't tell | Yes | Yes | Yes |
| Zhang & Chen 2008 ^9^ | Yes | Yes | Yes | Yes | Yes | No | Can't tell | No | No | Can't tell |

Abbreviation: CASP = Critical Appraisal Skills Programme (CASP) Qualitative Studies Checklist. ^10^

| **Table S4 Quality assessment results for the 43 cross-sectional studies via AXIS** | | | | | | | | | | | | | | | | | | | | |
| --- | --- | --- | --- | --- | --- | --- | --- | --- | --- | --- | --- | --- | --- | --- | --- | --- | --- | --- | --- | --- |
| First author and year | **Twenty assessing questions in the appraisal tool of AXIS** | | | | | | | | | | | | | | | | | | | |
|  | 1 | 2 | 3 | 4 | 5 | 6 | 7 | 8 | 9 | 10 | 11 | 12 | 13 | 14 | 15 | 16 | 17 | 18 | 19 | 20 |
| Cheng et al. 2018 ^11^ | Y | Y | N | Y | N | N | N | Y | Y | N | Y | Y | D | N | Y | Y | Y | Y | N | D |
| Fan& Che 2023^12^ | Y | Y | N | N | N | D | N | Y | Y | Y | N | N | N | N | Y | D | D | N | N | Y |
| Fu et al. 2011 ^13^ | Y | Y | N | Y | N | N | D | Y | N | Y | Y | Y | N | N | Y | Y | Y | Y | D | Y |
| Hao et al. 2023 ^14^ | Y | Y | Y | Y | N | D | N | Y | Y | Y | Y | Y | Y | N | Y | Y | Y | Y | N | Y |
| Hu et al. 2007 ^15^ | Y | Y | N | Y | N | D | D | Y | N | Y | Y | Y | D | N | Y | Y | Y | N | N | Y |
| Huang et al 2001 ^16^ | Y | Y | N | Y | D | Y | D | Y | Y | Y | Y | Y | D | N | Y | Y | Y | N | N | D |
| Ji et al. 2024^17^ | Y | Y | Y | Y | Y | Y | Y | Y | D | Y | D | Y | D | Y | Y | Y | N | Y | Y | Y |
| Jiang et al 2017 ^18^ | Y | Y | Y | Y | N | D | D | Y | Y | Y | Y | Y | N | N | Y | Y | Y | Y | N | Y |
| Jin et al 2015^19^ | Y | Y | N | Y | D | D | D | Y | Y | D | N | Y | D | N | Y | D | Y | N | N | D |
| Jin et al 2016 ^20^ | Y | Y | N | Y | D | D | D | Y | Y | N | Y | Y | D | N | Y | D | Y | N | N | D |
| Kazuma Sato 2020 ^21^ | Y | Y | Y | Y | Y | Y | D | Y | D | N | Y | Y | D | N | Y | Y | Y | Y | N | Y |
| Li et al 2019 ^22^ | Y | Y | N | Y | D | Y | D | Y | Y | Y | Y | Y | D | N | Y | Y | Y | Y | N | D |
| Lin et al 2024^23^ | Y | Y | D | Y | D | D | D | Y | D | Y | D | Y | D | N | Y | Y | D | Y | Y | Y |
| Lin & Huang 2010 ^24^ | Y | Y | N | Y | D | Y | D | Y | Y | N | Y | N | D | N | Y | Y | Y | N | D | D |
| Lin et al. 2021 ^25,26^ | Y | Y | Y | Y | N | Y | D | Y | Y | Y | Y | Y | N | N | Y | Y | Y | Y | N | Y |
| Lin&Lin 2016 | Y | Y | N | Y | D | D | D | Y | Y | Y | Y | N | D | N | Y | Y | Y | N | D | D |
| Liou et al 2008 ^27^ | Y | Y | D | Y | D | Y | D | Y | Y | Y | Y | Y | N | N | Y | Y | Y | Y | N | D |
| Liou et al. 2014 ^28^ | Y | Y | N | Y | D | D | D | Y | Y | Y | Y | Y | D | N | Y | Y | Y | Y | N | Y |
| Liu et al 2015 ^29^ | Y | Y | N | Y | D | Y | D | Y | Y | N | N | Y | D | N | Y | D | Y | N | D | D |
| Ma et al 2011 ^30^ | Y | Y | N | Y | D | Y | D | Y | Y | Y | D | Y | N | N | Y | Y | Y | N | D | D |
| Peng et al 2015 ^31^ | Y | Y | N | Y | N | Y | D | Y | Y | Y | N | Y | D | N | Y | D | Y | N | D | D |
| Shujin Lian 2012 ^32^ | Y | Y | N | Y | D | D | D | Y | Y | Y | N | N | D | N | Y | D | Y | Y | D | Y |
| Sun & Su 2005 ^33^ | Y | Y | N | Y | D | Y | D | Y | N | N | Y | Y | D | N | Y | Y | Y | N | D | D |
| Tanenbaum et al. 2017 ^34^ | Y | Y | N | Y | D | Y | D | Y | Y | Y | Y | Y | D | N | Y | Y | Y | Y | N | Y |
| Tao & Zhong 2010 ^35^ | Y | Y | N | Y | N | N | D | Y | Y | Y | Y | Y | N | N | Y | Y | Y | Y | D | Y |
| Tsou &Tsou 2003 ^36^ | Y | Y | N | Y | Y | Y | D | Y | N | Y | Y | Y | D | N | Y | Y | Y | Y | D | D |
| Wenjie Ling 2007 ^37^ | Y | Y | N | Y | D | D | Y | Y | N | N | N | N | N | Y | Y | D | Y | N | D | D |
| Wong & Huang 1999 ^38^ | Y | Y | N | Y | D | Y | N | Y | Y | Y | N | Y | D | N | Y | Y | Y | N | D | D |
| Worsley et al. 2017 ^39^ | Y | Y | N | Y | N | N | D | Y | N | Y | N | Y | Y | N | Y | Y | Y | Y | N | Y |
| Wu et al. 2022 ^40^ | Y | Y | N | Y | D | Y | D | Y | N | Y | Y | Y | D | N | Y | Y | Y | Y | N | Y |
| Xiang et al 2020 ^41^ | Y | Y | Y | Y | Y | Y | D | Y | Y | Y | Y | Y | D | N | Y | Y | Y | N | D | Y |
| Xingzhu Shen 2010 ^42^ | Y | Y | D | Y | D | Y | D | Y | N | D | Y | N | D | N | D | D | Y | N | D | D |
| Yan et al 2011 ^43^ | Y | Y | D | Y | D | Y | D | Y | N | Y | Y | Y | D | N | Y | Y | Y | Y | N | Y |
| Yang et al 2012 ^44^ | Y | Y | N | Y | D | N | D | Y | N | N | N | N | D | N | D | D | Y | N | D | D |
| Yao et al 2015 ^45^ | Y | Y | N | Y | D | D | D | Y | N | Y | N | Y | D | N | Y | D | Y | N | D | D |
| Yin et al 2017 ^46^ | Y | Y | N | Y | Y | D | D | Y | D | Y | N | N | D | N | Y | D | Y | N | D | D |
| Yunfei Lin 2009 ^47^ | Y | Y | N | Y | D | D | N | Y | N | Y | N | N | D | N | Y | D | Y | N | N | D |
| Zhang & Chen 2010 ^48^ | Y | Y | N | Y | D | D | D | Y | Y | Y | N | Y | D | N | Y | Y | Y | N | N | D |
| Zhang et al 2007 ^49^ | Y | Y | Y | Y | D | Y | D | Y | Y | Y | N | Y | D | N | Y | D | Y | N | D | D |
| Zhang et al 2008 ^50^ | Y | Y | N | Y | N | N | Y | Y | D | N | N | Y | N | Y | Y | D | Y | N | N | D |
| Zhang et al 2016 ^51^ | Y | Y | Y | Y | Y | Y | D | Y | D | Y | Y | Y | D | N | Y | Y | Y | N | D | D |
| Zhang et al.2022 ^52^ | Y | Y | N | Y | N | N | D | Y | Y | Y | Y | Y | D | N | Y | Y | Y | Y | N | Y |
| Zhou et al 2006 ^53^ | Y | Y | N | Y | Y | D | D | Y | Y | N | N | Y | D | N | Y | D | Y | N | D | D |

Abbreviations: AXIS= Appraisal tool for Cross-sectional Studies^54^; Don’t know = D; No = N; Yes = Y.

AXIS questions:

1 Were the aims/objectives of the study clear? (If you have comments, put into the "other" option. this function applies to all questions)

2 Was the study design appropriate for the stated aim(s)?

3 Was the sample size justified?

4 Was the target/reference population clearly defined? (Is it clear who the research was about?)

5 Was the sample frame taken from an appropriate population base so that it closely represented the target/reference population under investigation?

6 Was the selection process likely to select subjects/participants that were representative of the target/reference population under investigation?

7 Were measures undertaken to address and categorise Nn-responders?

8 Were the risk factor and outcome variables measured appropriate to the aims of the study?

9 Were the risk factor and outcome variables measured correctly using instruments/measurements that had been trialled, piloted or published previously? 10 Is it clear what was used to determined statistical significance and/or precision estimates? (e.g. p-values, confidence intervals)

11 Were the methods (including statistical methods) sufficiently described to enable them to be repeated?

12 Were the basic data adequately described?

13 Does the response rate raise concerns about Non-response bias?

14 If appropriate, was information about Non-responders described?

15 Were the results internally consistent?

16 Were the results presented for all the analyses described in the methods?

17 Were the authors' discussions and conclusions justified by the results?

18 Were the limitations of the study discussed?

19 Were there any funding sources or conflicts of interest that may affect the authors’ interpretation of the results

20 Was ethical approval or consent of participants attained?

**Reference**

1. Chang Y-J, Liou Y-M, Sheu S-J, Chen M-Y. Unbearable weight: young adult women's experiences of being overweight. *J Nurs Res*. Jun 2004;12(2):153-60. doi:10.1097/01.jnr.0000387498.47298.49

2. Chang L, Chattopadhyay K, Li J, Xu M, Li L. Interplay of support, comparison, and surveillance in social media weight management interventions: qualitative study. *JMIR Mhealth Uhealth*. Mar 1 2021;9(3):e19239. doi:10.2196/19239

3. Chu Y. 體重控制對女性大學生身體形象的意義(The meanings of weight-control toward female college students' body image). The Meanings of Weight-Control Toward Female College Students’ Body Image. *弘光學報*. 2008;(53):109-122. doi:10.6615/HAR.200808.53.09

4. Keyser-Verreault A. ‘Your fatty bum is really ugly!’ Gender, fat-shaming, and beauty-related tensions in contemporary Taiwanese families. *Journal of Family Studies*. 2023/09/03 2023;29(5):2068-2089. doi:10.1080/13229400.2022.2132984

5. Lee CF, Hsiung Y, Chi LK, Huang JP, Chen HH. "Help me fight my constant battle": A focus group study of overweight and obese women's mHealth app experiences to manage gestational weight gain. *Midwifery*. Jan 2023;116:103552. doi:10.1016/j.midw.2022.103552

6. Liou D, Bauer K. Obesity Perceptions among Chinese Americans. *Food, Culture & Society*. 2010/09/01 2010;13(3):351-369. doi:10.2752/175174410X12699432700908

7. Liou D, Bauer KD. Exploratory investigation of obesity risk and prevention in Chinese Americans. *J Nutr Educ Behav*. May-Jun 2007;39(3):134-41. doi:10.1016/j.jneb.2006.07.007

8. Mo X, Cao J, Tang H, Miyazaki K, Takahashi Y, Nakayama T. Inability to control gestational weight gain: an interpretive content analysis of pregnant Chinese women. *BMJ Open*. Dec 10 2020;10(12):e038585. doi:10.1136/bmjopen-2020-038585

9. Zhang J, Chen P. 中醫減重者之自覺健康研究調查(Research on self-perceptive health condition of people who use Traditional Chinese Medicine for weight loss). Research on Self-Perceptive Health Condition of People who Use TOM for Weight Loss. *台灣中醫臨床醫學雜誌*. 2008;14(2):116-122. doi:10.6968/TJCCM.200806.0116

10. Programme CAS. CASP for qualitative studies checklist. Internet. Jan 05, 2024. <https://casp-uk.net/checklists/casp-qualitative-studies-checklist-fillable.pdf>

11. Cheng MY, Wang S-M, Lam YY, Luk HT, Man YC, Lin C-Y. The relationships between weight bias, perceived weight stigma, eating behavior, and psychological distress among undergraduate students in Hong Kong. *The Journal of Nervous and Mental Disease*. 2018;206(9)

12. Fan S, Che B. 体重污名对肥胖大学生心理健康的影响及对策研究. The Impact of Weight Stigma on the Mental Health of Obese College Students and Countermeasures. *吉林体育学院学报*. 2023;39(3)

13. Fu TT, Lin YM, Huang CL. Willingness to pay for obesity prevention. *Econ Hum Biol*. Jul 2011;9(3):316-24. doi:10.1016/j.ehb.2011.02.003

14. Hao M, Yang J, Xu S, Yan W, Yu H, Wang Q. The relationship between body dissatisfaction, lifestyle, and nutritional status among university students in Southern China. *BMC psychiatry*. 2023;23(1):705. doi:<https://dx.doi.org/10.1186/s12888-023-05215-8>

15. Hu SP, Chiang TY, Yeh SL, Chien YW. Comparisons of attitudes and practices between obese and normal weight women in Taiwan. *Asia Pac J Clin Nutr*. 2007;16(3):567-71.

16. Huang G, Chen X, Duan F, Wen S. 超重和肥胖人群减肥方法及减肥意识调查(Survey on Weight Loss Methods and Awareness Among individuals affected by overweight and obesity.). *- 中国公共卫生*. - 2001-05-10 2001;- 17(- 5):- 431. doi:- 10.11847/zgggws2001-17-05-36

17. Ji L, Mu Y, Chang C, et al. Perceptions, attitudes and barriers to effective obesity care among people living with obesity and healthcare professionals in China: The ACTION-China study. *Diabetes, obesity & metabolism*. 2024;26(10):4694-4704. doi:<https://dx.doi.org/10.1111/dom.15837>

18. Jiang W, Tan J, Fassnacht DB. Implicit and explicit anti-fat bias among Asian females. *Eating and Weight Disorders - Studies on Anorexia, Bulimia and Obesity*. 2017/09/01 2017;22(3):457-465. doi:10.1007/s40519-016-0290-8

19. Jin B, Lian R, Tang D, Wang Y, Tan Q. 成都市323名公务员体重控制相关知识、行为现状分析 (Current status of weight control related knowledge and behaviours in 323 civil servants of Chengdu City). *预防医学情报杂志*. 2015;31(8):5.

20. Jin B, Cheng G, Lian R, Tang D. 成都市企业管理人员体重控制知信行现况分析(Knowledge attitude and practice of weight control in enterprise management personnel in Chengdu city). *预防医学情报杂志*. 2016;32(1):5.

21. Sato K. Unhappy and happy obesity: A comparative study on the United States and China. *Journal of Happiness Studies*. 2021/03/01 2021;22(3):1259-1285. doi:10.1007/s10902-020-00272-2

22. Li Y, Zhou P, Qiu L, Cheng X, Cao P, Zhang F. 海口市大学生肥胖相关知识、态度及行为调查(Survey on obesity-related knowledge, attitudes and behaviours among college students in Haikou City). *中国健康教育*. 2019;(012):035.

23. Lin Z, Si S, Liu J, et al. Unmet weight loss targets in real-world clinical practice: weight management and perceptions in China. *Frontiers in endocrinology*. 2024;15:1470394. doi:<https://dx.doi.org/10.3389/fendo.2024.1470394>

24. Lin S, Huang W. 大學生體型和健康自覺與飲食習慣之相關分析(A study of the dietary habit of college students with different body image and healthy consciousness). A Study of the Dietary Habit of College Students with Different Body Image and Healthy Consciousness. *人文與社會學報*. 2010;2(5):37-60. doi:10.30165/JHSS.201001.0001

25. Lin SW, Tsay SL, Lin KP. Prediction factors of weight control intention in Chinese young adults. *Int J Nurs Pract*. Jun 2021;27(3):e12927. doi:10.1111/ijn.12927

26. Lin H, Lin H. 体型认知，认知差距与大学女生运动参与程度及饮食态度 (The correlation between body shape perception, exercise participation and eating attitudes of female college students). *淡江体育学刊*. 2016;(19):51-63.

27. Liou TH, Huang N, Wu CH, Chou YJ, Liou YM, Chou P. Weight loss behavior in obese patients before seeking professional treatment in Taiwan. *Obes Res Clin Pract*. Mar 2009;3(1):1-52. doi:10.1016/j.orcp.2008.10.004

28. Liou D, Bauer K, Bai Y. Investigating obesity risk-reduction behaviours and psychosocial factors in Chinese Americans. *Perspect Public Health*. Nov 2014;134(6):321-30. doi:10.1177/1757913913486874

29. Liu X, Tang D, Tian H, Yang L, Tan S, Zhang X. 成都市四类职业人群对超重肥胖认知及体重控制行为调查(Status of overweight, obesity and weight control behaviours among 4 occupational populations in Chengdu City). *中国健康教育*. 2015;(4):408-410.

30. Ma C, Zhang L, Zhang Y, Lu M, Wang S. 新乡市在校女大学生减肥认知现状调查(Cognition situations of weight control among female college students in Xinxiang City). *中国学校卫生*. 2011;32(7):3.

31. Peng L, Li J, Huang Y, Li Y, Zhang L, Wang L. 武汉市大学生体重控制知信行调查 (Survey on weight management knowledge, beliefs, and practices among college students in Wuhan city). *公共卫生与预防医学*. 2014;25(3):3.

32. Lian S. 女大學生電視收視多寡、身體質量指數、自尊感、內化理想體型與身體意象研究（Television viewing, BMI, self-esteem, internalization and college women's perceptions of baby image）. Television Viewing, BMI, Self-Esteem, Internalization and College Women's Perceptions of Baby Image. *藝術學報*. 2012;(90):141-166. doi:10.6793/JNTCA.201204.0141

33. Sun J, Su H. 女大学生瘦身行为与认知现状的调查(Investigation on the current situation of college girls' behaviour and recognition of body slimming). *体育学刊*. 2005;12(5)

34. Tanenbaum HC, Felicitas JQ, Li Y, et al. Overweight perception: associations with weight control goals, attempts, and practices among Chinese female college students. *J Acad Nutr Diet*. Mar 2016;116(3):458-466. doi:10.1016/j.jand.2015.06.383

35. Tao Z, Zhong W. Eating attitudes and weight concern among Chinese middle-age women: A comparison between different age and BMI groups. *The European Journal of Psychiatry*. 2010;24(3):146-157. doi:10.4321/S0213-61632010000300003

36. Tsou M, Tsou M. 健康知識、教育程度與肥胖之關係 (The Relationship Between Health Literacy, Educational Attainment, and Obesity). Health Knowledge, Schooling, and Obesity. *台灣公共衛生雜誌*. 2003;22(4):295-307. doi:10.6288/TJPH2003-22-04-06

37. Ling W. 郑州市高校肥胖大学生减肥的调查研究(Survey and Research on Weight Loss Among College Students with obesity in Zhengzhou City). *现代预防医学*. 2007;34(7):3.

38. Wong Y, Huang Y-C. Obesity concerns, weight satisfaction and characteristics of female dieters: A study on female Taiwanese college students. *Journal of the American College of Nutrition*. 1999/04/01 1999;18(2):194-200. doi:10.1080/07315724.1999.10718850

39. Worsley A, Wang W, Sarmugam R, Pham Q, Februhartanty J, Ridley S. Family food providers' perceptions of the causes of obesity and effectiveness of weight control strategies in five countries in the Asia Pacific region: A cross-sectional survey. *Nutrients*. Jan 18 2017;9(1)doi:10.3390/nu9010078

40. Wu T, Liu W, Chen Y, Guo T, Sun R. The mediating effect of perceiving close relatives as obese on obesity and weight control behavior score among adults: An exploratory cross-sectional study in Chongqing, China. *Front Public Health*. 2022;10:984588. doi:10.3389/fpubh.2022.984588

41. Xiang J, Zhao T, Li X, Zhang J, Du X. 通江县15岁及以上常住居民超重肥胖危害知识知晓状况及影响因素分析（Analysis of Knowledge Awareness of the Hazards of Overweight and Obesity Among Residents Aged 15 and Above in Tongjiang County and Its Influencing Factors）. *预防医学*. 2020;(012):032.

42. Shen X. 甘肃省地方性高校女大学生减肥认知态度及行为调查（Survey on the Weight Loss Awareness, Attitudes, and Behaviors of Female College Students in Local Universities in Gansu Province）. *医学与社会*. 2010;(001):023.

43. Yan L, Chen X, Chen B, et al. 7039例不同体质指数成人肥胖相关知识、态度和行为调查(Study on obesity-related knowledge, attitude and behaviour in 7039 adults with different body mass index). *中国慢性病预防与控制*. 2011;19(5):4.

44. Yang Y, Xiao T, Xia K, et al. 超重及肥胖大学生自我健康意识及减肥行为的调查（The survey on self-awareness of health and weight loss behavior among undergradutes with overweight and obesity）. *中华医学教育杂志*. 2012;032(003):380-381,407.

45. Yao H, Liu Y, Guo X, et al. 女大学生减肥行为及其认知现状调查与分析(Investigation and analysis of female college students' losing-weight behaviours and their cognitive status). *中国妇幼健康研究*. 2015;26(4):3.

46. Yin F, Xiao R, Gong N, He F, Zhang Z, Hou Y. 云南某高校大学生BMI及减肥情况调查（Survey on BMI and Weight Loss Situation Among College Students at a University in Yunnan Province）. *中国食物与营养*. 2017;23(6):4.

47. Lin Y. 北方民族大学学生形态意象与肥胖认知的调查分析 (Survey and Analysis of Body Image Perception and Obesity Awareness Among Students of North Nationalities University). *宁夏大学学报（自然科学版）*. 2009;030(004):433-436.

48. Zhang P, Chen S. 肥胖人群的锻炼行为和锻炼动机-------以西安市是知识分子为例的比较研究(Exercise behavior and motivation among people with obesity: A comparative study of intellectuals in Xi'an city). 2010:

49. Zhang X, Zhao L, Yang Y. 肇庆市部分超重或肥胖机关干部人群的肥胖知信行现状（Survey on knowledge , attitude and behaviors about obesity among selected civil servants living with overweight or obesity in Zhaoqing）. *华南预防医学*. 2007;33(4):3.

50. Zhang X, Wu H, Zhang T. 女大学生对肥胖认知状况及减肥行为(Research and analysis of female undergraduates' fat cognition and weight-loss behaviour). *中国妇幼保健*. 2008;23(21):3.

51. Zhang L, Li J, Huang Y, et al. 武汉市18-55岁男性职业人群超重和肥胖相关知识知晓情况调查分析(Survey and analysis of knowledge awareness regarding overweight and obesity among male professionals aged 18-55 in Wuhan city）. *华南预防医学*. 2016;42(6):4.

52. Zhang W, Chen X, Wang C, Gao L, Chen W, Yang W. Perceptions and attitudes toward obesity and its management in migrants and rural residents in China: a cross-sectional pilot study. *Obes Surg*. Jan 2022;32(1):152-159. doi:10.1007/s11695-021-05755-6

53. Zhou W, Qiao L, Zhang L, Zhang H. 北京市东城区494名超重和肥胖成人健康状况及相关知识、态度、行为调查 (Survey on the health status and related KAP among 494 adults with overweight and obesity in Dongcheng District, Beijing City). *中国预防医学杂志*. 2006;7(2)

54. Downes MJ, Brennan ML, Williams HC, Dean RS. Development of a critical appraisal tool to assess the quality of cross-sectional studies (AXIS). *BMJ open*. 2016;6(12)
